# Functional consequences of calmodulin variants identified among schizophrenia patients and controls

**DOI:** 10.1038/s41398-025-03735-3

**Published:** 2025-11-22

**Authors:** Helene Halkjær Jensen, Malene Brohus, John W. Hussey, Ana-Octavia Busuioc, Emil Drivsholm Iversen, Faezeh Darki, Gabriela Dobromirova Nikolova, Amalie Elton Baisgaard, Palle Duun Rohde, Ida Elisabeth Gad Holm, Andrew McQuillin, Anders Olsen, Torben Moos, Ivy E. Dick, Michael Toft Overgaard, Mette Nyegaard

**Affiliations:** 1 Department of Chemistry and Bioscience, Aalborg University, Aalborg, Denmark; 2 Department of Pharmacology and Physiology, University of Maryland School of Medicine, Baltimore, MD USA; 3 Department of Health Science and Technology, Aalborg University, Aalborg, Denmark; 4 Department of Pathology, Aalborg University Hospital, Aalborg, Denmark; 5 Division of Psychiatry, University College London, London, UK; 6 Statens Serum Institut, Copenhagen, Denmark

**Keywords:** Molecular neuroscience, Schizophrenia

## Abstract

Calmodulin is a vital cellular calcium sensor expressed by the genes *CALM1-3*. Ultra-rare loss-of-function missense variants in the C-terminal lobe (C-lobe) of calmodulin are linked to cardiac arrhythmia, primarily long QT syndrome. Emerging evidence suggests calmodulin variants also contribute to neurological disorders. This study explores the allelic spectrum and functional consequences of calmodulin variants in schizophrenia. In a large-scale sequencing effort involving 24,248 schizophrenia patients and 97,322 control individuals, 25 unique calmodulin variants were found in 27 carriers, all ultra-rare. Seven carriers were schizophrenia patients, and 20 were controls. All patient variants affected the C-lobe, while only five of 20 control variants were in the C-lobe, linking C-lobe variants to increased schizophrenia risk. Functional analyses identified two variant classes in patients: Loss-of-function variants, reducing calcium affinity and impairing Ca_V_1.2 interaction, and gain-of-function variants, enhancing calcium affinity without affecting Ca_V_1.2 gating. Together, this study suggests that consequences of calmodulin variants include increased schizophrenia risk.

## Introduction

Calmodulin is a cellular calcium sensor. When calcium ions enter the cytosol as a cellular signal, calmodulin binds the ions and changes conformation, enabling calmodulin to bind and regulate hundreds of target proteins [1]. Calmodulin has four calcium binding sites organized in two lobes; the N-terminal lobe (N-lobe) and the C-terminal lobe (C-lobe). The C-lobe has approximately 10-fold higher calcium binding affinity than the N-lobe, whereas the N-lobe has faster kinetics than the C-lobe [1, 2]. Moreover, the N- and C-lobe play different roles in the interaction with downstream targets [3, 4], enabling calmodulin to tailor its molecular regulation mechanism to each interaction partner. Consequently, mutations in each of the two lobes can have vastly different physiological effects [5–7].

The protein sequence of calmodulin is exceptionally well-conserved through evolution, being identical in all vertebrates. Even lower animals such as *Drosophila melanogaster* and *Caenorhabditis elegans* carry only few amino acid differences compared to vertebrates [7–9]. In humans, calmodulin is encoded by three independent genes, *CALM1-3*, which produce identical calmodulin proteins [10].

Protein-altering human genetic variants in *CALM1-3* are linked to severe cardiac arrhythmias [1, 11, 12]. Since the initial discovery in 2012 [11], more than 140 cases of calmodulin variants have been identified in cardiac arrhythmia patients, most of whom suffer from long QT syndrome (LQTS) or catecholaminergic polymorphic ventricular tachycardia (CPVT) [11–13]. In 2023, *CALM1-3* were included in the genetic screening recommendations for LQTS and CPVT from the American College of Medical Genetics (ACMG) [14].

Calmodulin variants in cardiac patients are collected and described in the International Calmodulinopathy Registry (ICalmR) [13, 15]. While most of these patients have been identified after presenting with cardiac arrhythmia, increasing numbers of cardiac patients are also reported with neurological disorders. In the latest update of the ICalmR, 32% of the patients suffer from neurological features. While some of these may be secondary to a cardiac event, 20 patients (18%) present with primary neurological features such as intellectual disability, seizures, attention-deficit/hyperactivity disorder (ADHD), and autism spectrum disorder (ASD). Moreover, a single patient carrying an I64M variant is reported to have gross neurological and developmental defects, without any cardiac consequences [13]. Thus, these observations suggest that the phenotypic spectrum of calmodulinopathies also includes neurological disorders.

In these calmodulinopathy patients, almost all calmodulin variants are found in the C-lobe of the protein, thus there is a remarkable absence of N-lobe variants [3, 13]. Functional studies show that variants from LQTS patients impair calcium binding [1, 16] and affect calmodulin’s ability to regulate the voltage-gated calcium channel 1.2 (Ca_V_1.2) [12, 17–19].

Ca_V_1.2, encoded by the *CACNA1C* gene, is a plasma membrane voltage-gated calcium channel [20]. Calmodulin interacts with an IQ motif in the intracellular C-terminal of the channel. When Ca_V_1.2 opens in response to an action potential, calcium ions enter the cell, and calmodulin binds incoming calcium ions. This induces a conformational change that propagates to the Ca_V_1.2 channel and signals channel closure; a process called calcium-dependent inactivation (CDI) [21]. Calmodulin variants in LQTS patients reduce or abolish CDI [22]. In this way, a loss-of-function variant in calmodulin results in a gain-of-function effect on Ca_V_1.2 calcium flux [3].

Genetic variants in *CACNA1C* are associated with both cardiac and neurological disease. In the heart, variants in *CACNA1C* cause several cardiac arrhythmias, including short QT syndrome, Brugada Syndrome, and long QT syndrome type 8 (LQT8) [20]. In Timothy Syndrome patients, variants in *CACNA1C* cause LQT8 in combination with developmental defects, syndactyly, and ASD [23, 24]. *CACNA1C* variants have also been found in patients who present with neurological disorders alone, including developmental delay, epilepsy, and intellectual disability [20, 25]. Finally, genetic variants in *CACNA1C* have frequently been identified as genetic risk factors in genome-wide association studies of schizophrenia (SCZ) [20, 26–28]. While effects on Ca_V_1.2 conductance and inactivation have been observed for some of these neurological variants, effects on CDI remain unknown [24, 25, 29, 30].

Although calmodulin variants have only recently been suggested as contributors to neurological diseases [9, 13, 31], there is a broad biological context to understanding a pathological role of calmodulin variants in the brain: Calmodulin is a key regulator of numerous ion channels expressed in neurons (e.g. Ca_V_ and Na_V_ isoforms), receptors (e.g. NMDA and IP_3_ receptors), kinases (e.g. CaMKII), and phosphatases (e.g. CaN and PDE). As such, calmodulin modulates neuronal development, health, and synaptic firing [32, 33]. Interestingly, a number of anti-psychotic agents directly interact with calmodulin or affect the calmodulin signaling axis [34–36], and reduced calmodulin expression has been reported in *post mortem* brains of SCZ patients [37–39].

Inspired by the neurological roles of calmodulin, we explored the allelic spectrum of calmodulin variants in SCZ. We accessed a recently published exome sequencing study of 24,248 SCZ patients and 97,322 control individuals [40]. In this population, we found seven SCZ patients with calmodulin missense variants, all in the C-lobe of the protein. In the control group, we found 20 carriers of missense variants, distributed throughout the protein. These SCZ and control variant distribution patterns were significantly different from each other, and C-lobe variants were statistically associated with increased SCZ risk. We functionally assessed the effects of the SCZ and control variants on the calcium affinity of calmodulin and the interaction with and regulation of Ca_V_1.2. We found that SCZ variant effects separated into gain-of-function or loss-of-function effects that were generally of smaller magnitude than effects reported for LQTS variants, but larger than for variants from control individuals.

## Materials and methods

### Identification of *CALM* missense variants in SCHEMA

The Schizophrenia Exome Sequencing Meta-analysis (SCHEMA) has collected full exome or whole genome sequences from 11 international studies of patients diagnosed with schizophrenia or schizoaffective disorder [40]. Control subjects were recruited from the same studies as well as from non-psychiatric and non-neurological collections in the Genome Aggregation Database (gnomAD). Control individuals in SCHEMA are thus a combination of individuals with no known psychiatric diagnosis and individuals selected from other population registries. Summary-level data from SCHEMA is available through https://schema.broadinstitute.org. Here, we searched for the genes *CALM1*, *CALM2*, and *CALM3* (last accession on 22^nd^ of September 2023). In the resulting list of genetic variants, we filtered for “missense only”. For all missense variants, we extracted information of their position in the gene, the character of the variant, MPC and CADD score (available when clicking on each variant), and whether they were observed in the patient population or in the control population. Other participant information is not available through the browser, and the data is effectively anonymized.

### GTEx accession

To assess *CALM1-3* gene expression across brain regions, we accessed the Genotype-Tissue Expression (GTEx) portal V8 [41]. To extract short read RNA sequencing data, we used the multi gene query through https://www.gtexportal.org/home/multiGeneQueryPage, last on 30^th^ October 2023. Here, we searched for the genes *CALM1, CALM2, CALM3, CACNA1C, CACNA1G, CLDN4, CUL1, GRIA3, GRIN2A, HERC1, RB1CC1, SETD1A, SP4, TRIO, UBC*, and *XPO7*. In the search, we included all available brain regions (amygdala, anterior cingulate cortex (BA24), caudate (basal ganglia), cerebellar hemisphere, cerebellum, cortex, frontal cortex (BA9), hippocampus, hypothalamus, nucleus accumbens (basal ganglia), putamen (basal ganglia), spinal cord (cervical c-1), and substantia nigra), lung (an epithelial tissue), heart left ventricle, and skeletal muscle. We extracted the median transcript-per-million (TPM) for all genes in all tissues.

### Human brain tissue

We used brain samples containing hippocampal tissue from two adults (63 y and 50 y, both male) with no reported neurological disorders. The tissue was fixed in 4% paraformaldehyde for four weeks and embedded in paraffin.

### Consent to participate

Tissue samples were obtained from autopsy following written informed consent by the next of kin, according to the Danish legislation.

### RNA in situ hybridisation

The brain tissue was cut on a microtome at 3 µm thickness and de-paraffinated in Neo-Clear (109843, Merck). Deparaffination and in situ hybridization was performed using the RNAscope® 2.5 HD Detection Reagent RED kit (322360, Advanced Cell Diagnostics), following the manufacturer’s recommendations. Hybridization was performed for 2 h at 40 °C in a HybEZ™ II Hybridization Oven (321720, Advanced Cell Diagnostics) with probes to detect *CALM1* (461121), *CALM2* (499931), *CALM3* (1128281), *UBC* (positive control, 312028), and *DapB* (negative control, 310043) (all Advanced Cell Diagnostics). Following development of the Fast Red chromogenic signal, slides were counterstained with Mayer’s hematoxylin (MHS16, Merck) for 2 min. Slides were airdried for 15 min at 60 °C before mounting in VectaMount® Permanent Mounting Medium (H-5000, Vector Laboratories). Imaging was performed on a Hamamatsu NanoZoomer-S360 Visiopharm slide scanner with identical settings for all images. Image analysis and preparation of figures were performed in QuPath [42] and FIJI/ImageJ (freely available from NIH). No post-exposure adjustments were made to the images.

### Single-cell transcriptomics analysis

To assess *CALM1-3* gene expression in prenatal brain, we analyzed the single-cell gene expression profile of the entire left hippocampus during gestational weeks 16-27 [43]. The data is accessible in the Gene Expression Omnibus (GEO) under accession number GSE131258. Data processing follows the guidelines described [43]. Briefly, the gene-cell data matrix was obtained from GEO website. The most recent version of the R Seurat package (v4.3.0) was used for data analysis. Poor-quality cells with expression of fewer than 800 genes and more than 7000 genes were excluded from the dataset. The genes expressed in at least 30 single cells were entered in the analysis. Hemoglobin-expressing cells and those with more than 15% mitochondrial gene expression were also eliminated. In total, 42954 single cells expressing 17737 genes were included in the downstream analysis. The data underwent normalization for sequencing depth to achieve a total of 10^4^ molecules per cell. Cell types were identified using the top highly variable genes and by the expression of known cell-type markers. Average gene expression levels for the genes of interest were calculated in the Seurat package for all cells in each cluster and for the cells with expression value above zero for the consequent genes for better visualization. The ggplot2 package was used to plot expression patterns across the cell types.

### Calmodulin protein production

Calmodulin variants were expressed in *Escherichia coli* Rosetta 2 (DE3), from a modified pET vector, as fusion proteins carrying an N-terminal maltose-binding protein (MBP) followed by a tobacco etch virus (TEV) cleavage site. Transformed bacteria were grown in LB medium (10 g/l NaCl, 5 g/l yeast extract, 10 g/l tryptone, pH 7.5) at 37 °C until reaching the exponential growth phase, then overnight protein expression was induced at 25 °C by addition of 1 mM isopropyl β-D-1-thiogalactopyranoside (IPTG).

Bacterial cells were harvested by centrifugation (6000 x *g*, 10 mins) and lysed by sonication followed by another centrifugation step (40,000 x *g*, 45 mins) to obtain the lysate. The lysate was filtered (0.2 µm) and applied to a custom packed amylose column (New England Biolabs) attached to an NGC Chromatography System (Bio-Rad). After sample application, the column was washed with three column volumes (CV) of buffer A (20 mM Tris, 100 mM NaCl, 1 mM EDTA, 1 mM DTT, pH 7.4) after which the protein was step eluted with 100% buffer B (buffer A added 10 mM maltose).

The MBP-calmodulin fusion protein was cleaved using TEV protease (1 mg TEV:100 mg fusion protein) overnight at 4 °C. To separate MBP from calmodulin, the sample was applied to a 20 ml Q sepharose FF (Cytiva) column attached to an NGC Chromatography System (Bio-Rad). After sample application, the sample tubing was rinsed with buffer A (20 mM Tris, 100 mM NaCl, pH 7.4), before washing the column with 4 CV 35% buffer B (20 mM Tris, 500 mM NaCl, pH 7.4). Calmodulin was step eluted with 70% buffer B.

The calmodulin-containing fractions were pooled and concentrated to ~2.5 ml using a 10,000 molecular-weight cut-off centrifugal filter in preparation for a final size exclusion chromatography (SEC) step. After concentration, 20 mM EDTA was added to the sample to remove residual calcium ions before applying the sample to a HiLoad 16/600 Superdex 75 pg column (Cytiva) attached to an ÄKTA Purifier chromatography system. The protein was eluted in HK buffer (20 mM HEPES, 100 mM KCl, pH 7.2). The integrity and purity of the calmodulin protein was verified by SDS-PAGE (Fig S1A) and the final product was aliquoted and stored at -80 °C until further analysis. The identity of the purified calmodulin variants was verified by MALDI-TOF.

### Calcium/magnesium-buffered solutions

The free calcium concentration was controlled using a dual-chelator buffer system (50 mM HEPES, 100 mM KCl, 2 mM NTA, 0.5 mM EGTA, pH 7.2) [44]. Practically, three individual buffers were prepared, all with the previously mentioned composition of chemicals at 1.5x their final concentration. Additionally, one of the three buffers was supplemented with 3 mM calcium (1x), and another was supplemented with 30 mM magnesium (1x). The three buffers (no calcium, high calcium, and high magnesium) were mixed in different ratios to obtain a free magnesium concentration of 1 mM and range of desired free calcium concentrations, calculated using a pCa calculator [44]. For calcium titration experiments, a fourth buffer was prepared by spiking the 3 mM calcium buffer to 12.5 mM (1x) with 1 M CaCl_2_.

### Calcium binding to calmodulin

Calcium/magnesium buffers (see above) were mixed to obtain a free magnesium concentration of 1 mM and a range of free calcium concentrations, from 40 nM to 8 mM, using an automated Microlab STARlet liquid handling robot (Hamilton). Calmodulin (15 µM) and TCEP (300 µM, 20x [calmodulin]) was added during dilution of the buffers to 1x.

16 discrete calcium/calmodulin solutions were prepared and sequentially transferred to a quartz cuvette. Changes in the intrinsic phenylalanine and tyrosine fluorescence, reflecting calcium binding to the calmodulin N-lobe and C-lobe, respectively, were monitored using a FluoroMax-4 spectrofluorometer (Horiba scientific). For phenylalanine fluorescence, the excitation wavelength was 250 nm (slit 8 nm) and the emission wavelength was 280 nm (slit 8 nm). For tyrosine fluorescence, the excitation wavelength was 277 nm (slit 5 nm) and the emission wavelength was 310 nm (slit 5 nm). Three (tyrosine) or four (phenylalanine) technical recordings were averaged for at least three individual experiments.

The fluorescence intensity (FI) was plotted as a function of the free calcium concentration ([Ca^2+^_free_]) to generate a calcium binding curve. A generic Hill model, considering cooperative calcium binding via the Hill coefficient, h, was fitted to determine the apparent calcium binding affinity of calmodulin via the dissociation constant, K_D,app_:

$${FI}={{FI}}_{{span}}\cdot \frac{{\left[{{Ca}}_{{free}}^{2+}\right]}^{h}}{{\left[{{Ca}}_{{free}}^{2+}\right]}^{h}+{{K}_{D,{app}}}^{h}}+{{FI}}_{{initial}}$$

Non-linear curve fitting, according to this model, was done using GraphPad Prism. The dissociation constants were log_10_-transformed before statistical comparison using one-way ANOVA with Dunnett’s *post hoc* test for multiple comparisons.

### Secondary structure determination by circular dichroism

Circular dichroism spectra were recorded at 25 °C using a Chirascan Plus spectrometer (Applied Photophysics) for 20 µM calmodulin in a buffer consisting of 4 mM HEPES, 20 mM KCl, 100 µM TCEP, and either 1 mM EDTA or 1 mM CaCl_2_. Spectra were recorded at 190-260 nm with a bandwidth of 1 nm, a step size of 0.5 nm, and a sampling time-per-point of 0.5 seconds. Each spectrum represents the average of two technical recordings for at least four individual measurements. After data collection, each spectrum was buffer subtracted and the signal was converted to mean residue ellipticity, θ_MRE_.

As a spectral descriptor of structural changes caused by calmodulin variants, we calculated the θ signal ratio at 222 and 208 nm, θ_222/208_, reflecting changes in the structure and environment of alpha-helices (highly relevant for calmodulin which consists primarily of alpha-helices both in its apo- and calcium-bound state). Furthermore, as a spectral descriptor of structural changes in the transition from the apo to calcium-bound form, we calculated the change in θ signal at 222 nm, by subtracting the calcium-spectrum signal from the apo-spectrum signal, and dividing it by the apo-spectrum signal, Δθ_222_/θ_222_. Statistical testing for structural differences from the WT was done using one-way ANOVA and Dunnett’s *post hoc* test for multiple comparisons.

### Calcium-dependent calmodulin binding to the Ca_V_1.2 IQ-domain

The interaction between calmodulin and the IQ-domain from Ca_V_1.2 was monitored by changes in the fluorescence anisotropy (FA) of a 5-TAMRA-labeled human Ca_V_1.2-IQ peptide (DEVTVGKFYATFLIQEYFRKFKKRKEQGLVGKPS) during titration with calmodulin [45]. Calcium/magnesium buffers (see above) were mixed to obtain a free magnesium concentration of 1 mM and a range of free calcium concentrations, from 3 nM to 400 µM, using an automated Microlab STARlet liquid handling robot (Hamilton). The TAMRA-labeled peptide ( ~ 20 nM) was added during dilution of the buffers to 1x. Using the liquid handling robot, 3 µl calmodulin was added to the first column of a 384-well plate (Corning) followed by 65 µl calcium buffer/peptide solution. For the four lowest calcium concentrations, a ~ 600 µM calmodulin stock was used, whereas for the four highest calcium concentrations, a 22.7 µM calmodulin stock was used. Then, 30 µl calcium buffer/peptide solution was aliquoted into the remaining wells in the plate, resulting in one specific calcium concentration per row. Finally, calmodulin was serial diluted by column-wise transferring 38 µl liquid and discarding the last 38 µl. Half a 384-well plate was used for each calmodulin variant, resulting in eight calmodulin binding curves at eight different calcium concentrations [45]. The FA signal was measured at 25 °C in a Spark plate reader (TECAN) using a 535 (25) nm excitation filter and a 590 (20) nm emission filter, a 560 dichroic mirror, 20 flashes, a 40 µs integration time, a 200 ms settling time, a gain of 82, and a z-height of 19740 µm. A G-factor of 0.99 was determined using free 5-TAMRA.

For each of the eight binding curves, a stoichiometric binding model was fitted:

$${FA}={{FA}}_{{span}}\cdot \left(\frac{{{\rm{K}}}_{{\rm{D}}}+\left[\mathrm{IQ\; domain}\right]+\left[\mathrm{CaM}\right]}{2\cdot \left[\mathrm{IQ\; domain}\right]}-\sqrt{{\left(\frac{{{\rm{K}}}_{{\rm{D}}}+\left[\mathrm{IQ\; domain}\right]+\left[\mathrm{CaM}\right]}{2\cdot \left[\mathrm{IQ\; domain}\right]}\right)}^{2}-\frac{\left[\mathrm{CaM}\right]}{\left[\mathrm{IQ\; domain}\right]}}\right)+{{FA}}_{{initial}}$$

Where K_D_ is the dissociation constant, and [IQ domain] and [CaM] is the total concentration of the 5-TAMRA-labeled IQ peptide and calmodulin, respectively. Non-linear curve fitting, according to this model, was done using GraphPad Prism v6.07. At least three independent experiments were performed for each calmodulin variant. The dissociation constants were log_10_-transformed before statistical comparison using one-way ANOVA with Dunnett’s *post hoc* test for multiple comparisons at each of the eight calcium concentrations.

A plot of the log_10_ transformed K_D_-values as a function of the log_10_ transformed free calcium concentration resulted in a sigmoidal calcium-dependent affinity curve. As a measure of the calcium sensitivity of the calmodulin:Ca_V_1.2-IQ interaction, a variable slope EC_50_ model was fitted to these curves:

$$log\,{K}_{D}=log\,{K}_{D,{span}}\bullet \left(\frac{1}{1+{\left(\frac{{EC}50}{log\left[{{Ca}}_{{free}}^{2+}\right]}\right)}^{{slope}}}\right)+log\,{K}_{D,{initial}}$$

Statistical comparison of EC_50_ values was done using one-way ANOVA with Dunnett’s *post hoc* test for multiple comparisons.

### Preparation of HEK293 Cells for Whole-Cell Patch Clamp

HEK293 cells (CRL1573, ATCC) were employed for electrophysiological experiments. The cells were routinely tested for mycoplasma. For experiments, HEK293 cells were cultured in 10-cm dishes on glass coverslips and transfected using an established calcium phosphate method [46, 47]. 8 μg each of human α_1C_ cDNA, rat brain β_2a_ (M80545), rat brain α_2_δ (NM_012919.3) subunits, and WT or variant calmodulins were heterologously co-expressed. Expression was further increased by co-transfection with 2 μg of simian virus 40 T antigen (Tag) cDNA. The α_1C_ plasmid encoding a human *CACNA1C* (Ca_V_1.2) channel variant contains exon 8a, within a pcDNA3 expression vector (Acc# Z34810) [24]. The WT calmodulin construct was the human *CALM1* cDNA [18] within the pIRES2-EGFP vector (Clontech Laboratories). From this WT calmodulin-pIRES construct, pathogenic calmodulin variants were generated using QuikChange Lightning™ site-directed mutagenesis (Agilent). Expression of all constructs was driven by a cytomegalovirus (CMV) promoter. Calmodulin variants were overexpressed compared to endogenous levels (Fig S1B), and plasmid expression was verified visually by the expression of EGFP prior to all experiments.

### Electrophysiological Preparations

Whole-cell voltage-clamp recordings of HEK293 cells were carried out 24-48 h post-transfection at ambient room temperature. An Axopatch 200B amplifier (Axon Instruments) was used to obtain recordings. These recordings were lowpass filtered at 2 kHz and digitally sampled at 10 kHz to minimize noise. Series resistances of 1.5-4 MΩ were utilized, as well as P/8 leak subtraction. Internal solutions contained (in mM): CsMeSO_3_, 114; CsCl, 5; MgCl_2_, 1; MgATP, 4; HEPES (pH 7.4), 10; and BAPTA, 10; at 295 mOsm adjusted with CsMeSO_3_. External solutions contained (in mM): TEA-MeSO_3_, 140; HEPES (pH 7.4), 10; and CaCl_2_ or BaCl_2_, 40; at 300 mOsm, adjusted with TEA-MeSO_3_. These solutions produced the following uncorrected junction potentials: 10 BAPTA/40 Ca^2+^: 10.5 mV; 10 BAPTA/40 Ba^2+^: 10.2 mV [48]. The fraction of peak current after a 300-ms step depolarization (*r*_*300*_) at test voltages was measured. Ca^2+^/calmodulin-dependent inactivation (CDI) was evaluated as *f*_*300*_ = (Ba *r*_*300*_ – Ca *r*_*300*_).

### Western blotting

HEK293 cells were cultured in 6 well-plates. Subsequently, the cells were transfected with or without plasmids containing calmodulin WT or calmodulin mutants as described above [47]. 48 h post-transfection, the samples were lysed using RIPA lysis and extraction buffer with a protease inhibitor to minimize protein degradation. Total protein concentration was evaluated with a BCA assay (#23225, Thermo Fisher) to ensure equal per-sample loading. Samples were run in an SDS-PAGE gel at 70 V for 10 min, then 170 V for 30 min. The resultant gels were blotted onto a methanol-activated PVDF membrane at 75 V for 1 h. The membrane was blocked in 3% BSA and then incubated with an anti-calmodulin antibody (Ab124742, Abcam) at 1:5000 or anti-β-actin antibody (#4967 L, Cell Signaling) at 1:3000, followed by secondary antibody (#7074S, Cell Signaling) at 1:3000. Primary antibodies were diluted in blocking solution and incubated for 16 h at 4 °C, secondary antibodies were incubated at room temperature for 1 h. The membrane was treated with stripping buffer (#21059, Thermo Fisher) between the calmodulin and actin blots. Visualization of the membranes was performed with a chemiluminescent dye kit (#34580, Thermo Fisher) and a ChemiDoc imager (Bio-Rad).

### Statistical analyses

Statistical analysis was performed with GraphPad Prism or Mathworks MATLAB using indicated methods. There was similar variance between groups being statistically compared. The significance levels are given as: *P < 0.05, ** *P* < 0.01, *** *P* < 0.001, **** *P* < 0.0001. Logistic regression was used to test for association between calmodulin missense carrier status and schizophrenia disease status. The association test was based on the asymptotic assumption that the likelihood ratio test (${LR}=-2\log \frac{L({m}_{f})}{L({m}_{r})}$), where $L({m}_{f})$ is the likelihood of the full model including the variant status, while $L({m}_{r})$ is the likelihood of the reduced model neglecting the variant status) follows a ${\chi }^{2}$ distribution with one degree of freedom [49].

### Preparation of plots and figures

Data handling was performed using Microsoft Excel, GraphPad Prism, R, and Mathworks MATLAB. Microscope images were prepared in QuPath and FIJI ImageJ. Figures were prepared in GraphPad Prism, Mathworks MATLAB, and Inkscape.

## Results

### Calmodulin C-lobe variants associate with increased SCZ risk

**Fig. 1:**
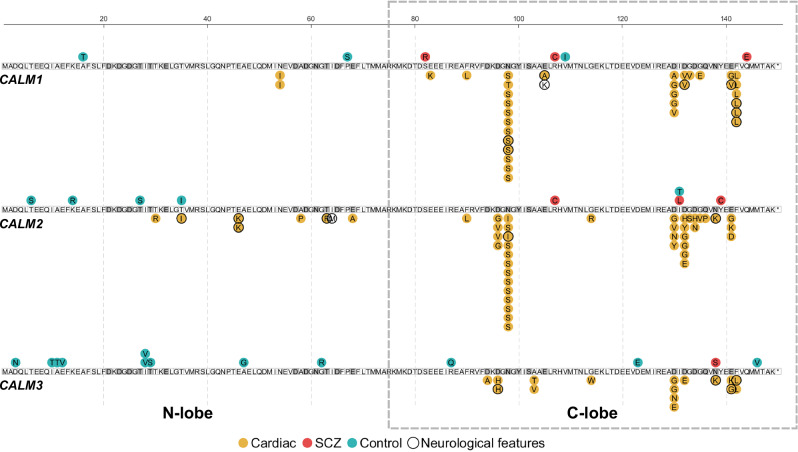
*CALM1-3* protein variants in cardiac and SCZ patients are enriched in the calmodulin C-lobe. Three genes, *CALM1-3*, produce identical calmodulin proteins, represented by the three rows. Numbering follows the immature 149 amino acid sequence, although the initial methionine is cleaved off during translation. Each circle represents an independent case of a calmodulin variant carrier. Circles below the sequences represent patients reported in the ICalmR, and circles above represent patients identified in the present study. Colors of the circles reflect carriers with cardiac arrhythmia (yellow), SCZ (red), or control carriers (teal), with the associated amino acid position and substitution indicated. Carriers from the ICalmR with primary neurological features are indicated with a black circle. The C-lobe of calmodulin, harboring the majority of cardiac and all SCZ variants, is framed with a dashed square. Amino acids that coordinate calcium are highlighted in dark gray. *ICalmR: International Calmodulinopathy Registry.* *LQTS: Long QT syndrome. SCZ: schizophrenia*.

The Schizophrenia Exome Sequencing Meta-Analysis (SCHEMA) contains exomes from 24,248 patients with schizophrenia or schizoaffective disorder and from 97,322 control individuals [40]. In this collection, we found in total 27 carriers of missense variants in *CALM1-3* (Fig. 1, Table S1). Seven of these carriers were patients with schizophrenia (SCZ) (carrier frequency ~1:3500), while the remaining 20 carriers were observed in the control population (carrier frequency ~1:4900).

There was no evidence for enrichment of missense variants in SCZ in the calmodulin genes (odds ratio (OR) 1.40, 95% confidence interval (CI) 0.55-3.2, Table 1). Because the calmodulin N-lobe and C-lobe are functionally different, we tested if the variants were equally distributed between the two lobes in SCZ cases and controls. In the calmodulin C-lobe we observed seven missense variants in SCZ patients (0.029%) and five missense variants in control subjects (0.0051%), resulting in an increased SCZ risk (OR 5.62, 95% CI 1.8-19). In contrast, for the calmodulin N-lobe, we observed no SCZ subjects carrying a missense variant, whereas 15 control subjects carried a missense variants (Table 1, Fig. 1).

**Table 1:** Odds ratios for *CALM1-3* variants in SCZ cases and controls.

	SCZ *CALM* carriers (n = 24,248)	Control *CALM* carriers (n = 97,322)	OR	95% CI lower	95% CI upper	*P*-value	*P*_*adj*_-value*
***CALM1*** + ***2*** + ***3***	7	20	1.40	0.55	3.2	0.52	0.57
N-lobe	0	15	3.80 E-05	NA	0.010	0.0098	0.054
C-lobe	7	5	5.62	1.8	19	0.0036	0.043
***CALM1***	3	3	4.01	0.74	22	0.10	0.20
N-lobe	0	2	2.80 E-04	NA	14	0.35	0.42
C-lobe	3	1	12.0	1.5	0.24 E + 03	0.018	0.054
***CALM2***	3	5	2.40	0.49	9.8	0.25	0.33
N-lobe	0	4	2.80 E-04	NA	0.51	0.18	0.31
C-lobe	3	1	12.0	1.5	0.24 E + 03	0.018	0.054
***CALM3***	1	12	0.330	0.020	1.7	0.22	0.33
N-lobe	0	9	1.00 E-04	NA	0.040	0.045	0.11
C-lobe	1	3	1.34	0.070	10	0.81	0.81

* False discovery rate (FDR)-adjusted *P*-values. *CI* confidence interval. *OR* odds ratio. *SCZ* schizophrenia.

The pathogenicity of genetic variants can be predicted using different computational tools. The SCHEMA study uses the MPC pathogenicity score [50], where MPC ≥ 2 is considered pathogenic. Of the total 27 calmodulin variant carriers, eight carried a variant with an MPC score ≥2. Of these eight carriers, four were SCZ patients with variants that affect the C-lobe, and four were control individuals with variants in the N-lobe (Table S1). When restricting to carriers of variants with MPC score ≥2, the association of C-lobe variants with SCZ had the same effect size but the *P*-value became lower despite fewer variants, suggesting that the association signal came from variants with MPC ≥ 2 (Table S2).

We also noted a different distribution of SCZ and control variants between the three genes (Fig. 1). Of the seven SCZ patients, three carried variants in *CALM1*, three carried variants in *CALM2*, and one had a variant in *CALM3*. On the contrary, of the five C-lobe variant carriers in the control group, one had a variant in *CALM1*, one had a variant in *CALM2*, and three carriers had variants in *CALM3*. Therefore, we evaluated the contribution of each *CALM* gene to the increased SCZ risk associated with calmodulin C-lobe variants (Table 1). Although not significantly different, C-lobe variants result in a different pattern of risk association for *CALM1* and *CALM2* (both OR 12.0, 95% CI 1.5-243) compared to *CALM3* (OR 1.3, 95% CI 0.070-10).

Together, these observations demonstrated an increased SCZ risk from calmodulin C-lobe variants and indicated that most of this risk came from *CALM1* and *CALM2*. Of note, calmodulin variants in cardiac patients mainly affect the C-lobe [13], emphasizing the importance of considering the two lobes separately when assessing effects of missense variants. It is also worth noting that we found no identical variants between cardiac patients, SCZ patients, and control individuals.

### *CALM1*, *CALM2*, and *CALM3* are highly expressed in the brain

**Fig. 2:**
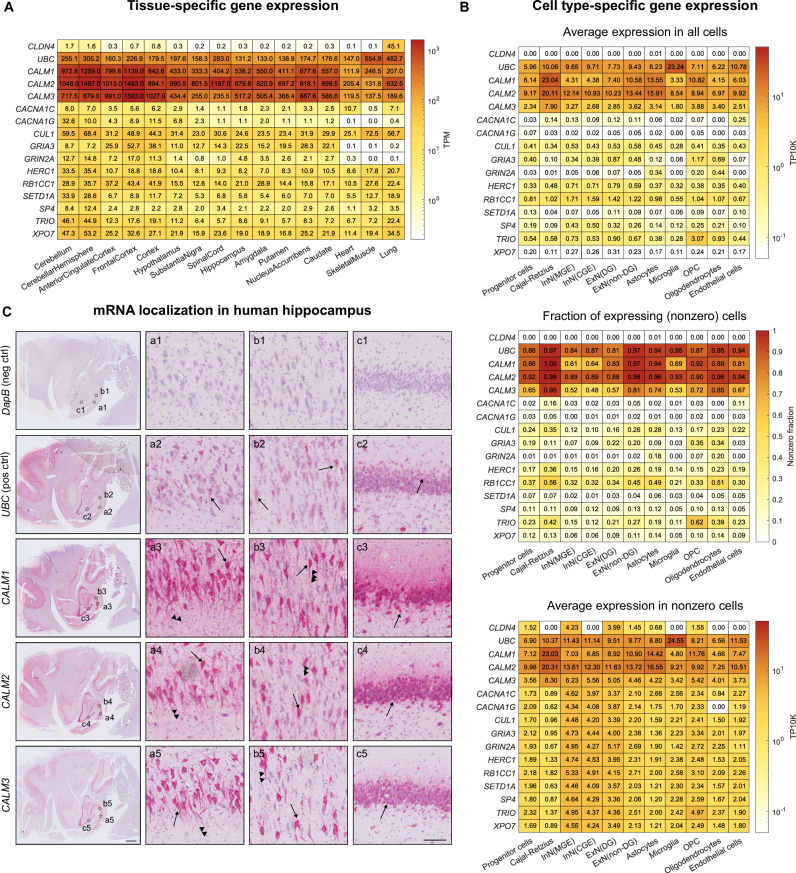
High levels of *CALM1-3* mRNA in the brain. **A**, **B** mRNA levels of *CALM1-3*, *CLDN4* (negative control), *UBC* (positive control), *CACNA1C* (encodes Ca_V_1.2), and ten genes for which rare genetic variants are associated with increased SCZ risk [40]. **A** Transcript levels extracted from the genotype-tissue expression (GTEx) project. **B** Single-cell spatial transcriptomics data from human hippocampal fetuses at gestational weeks 16-27 [43] (see also Figs. S2–4). **C** In situ hybridization with RNAscope probes (red signal) targeting the indicated genes of a healthy human donor. Arrows indicate examples of positively stained neuronal cell bodies, and arrowheads indicate examples of stained neuronal processes. The images are representative of three individual stains of two donors (see also Fig. S5). Scale bars are 2 mm and 100 µm for insets. *Ca*_*V*_*1.2: Voltage-gated calcium channel isoform 1.2. ExN(DG): Dentate gyrus excitatory neurons. ExN(non-DG): Non-dentate gyrus excitatory neurons. InN(CGE): Caudal ganglion eminence-derived inhibitory neurons. InN(MGE): Medial ganglion eminence-derived inhibitory neurons. OPC: oligodendrocyte progenitor cells. SCZ: schizophrenia. TP10K: Transcripts per 10,000. TPM: Transcripts per million*.

Calmodulin protein levels are high in the brain ( ~ 0.5 mg/g) [10], but since the protein product of the *CALM1*, *CALM2*, and *CALM3* genes is identical, it cannot be easily determined if the protein is produced from all three genes. Because the variant pattern in *CALM3* stood out from that in *CALM1* and *CALM2*, we investigated if all three genes are expressed in the brain. To study the *CALM* expression level in different brain regions, we used the Genotype-Tissue Expression (GTEx) database [41]. For comparison, we included heart left ventricle, skeletal muscle, and lung tissues. As a low expression control gene, we included *CLDN4*, which encodes claudin 4 and is primarily expressed in epithelia, including the lung [51] (Fig. 2A, first row). As a high expression control gene, we included *UBC*, which encodes polyubiquitin C and is highly expressed in all tissues [52, 53] (Fig. 2A, second row). For reference, we also included *CACNA1C* and ten other genes (*CACNA1G, CUL1, GRIA3, GRIN2A, HERC1, RB1CC1, SETD1A, SP4, TRIO, XPO7*), for which rare genetic variants have been associated with increased SCZ risk in the SCHEMA study [40]. Strikingly, all three *CALM* genes had the highest expression level across all brain tissues and genes investigated, even higher than that of the *UBC* positive control (Fig. 2A).

We further explored the expression patterns of these genes in a single-cell spatial transcriptomics sequencing dataset from human fetal hippocampus [43]. Supporting our findings on the tissue-level, *CALM1*, *CALM2*, and *CALM3* expression levels were high and similar to the positive control across all hippocampal cell types (Fig. 2B, upper panel) and across all gestational stages (Figs. S2–4). During analysis, we noticed that this dataset – like other spatial transcriptomics datasets – included a subset of cells with zero *CALM* gene expression [54] (Figs. S2–4). It is unclear whether the presence of *CALM*-zero cells is a biological finding or a technical issue [54]. Therefore, we determined the expression levels in the non-zero cells population only (Fig. 2B, middle and bottom panels). First, we calculated the fraction of non-zero-to-total number of cells and found that *CALM1* and *CALM2* expression was measured in 61-100% of the cells, whereas *CALM3* expression was measured in 48-95% (Fig. 2B, middle panel). When considering expression levels only in non-zero cells, we consistently found that all three *CALM* genes were expressed across the brain at levels similar to or higher than the included control and reference genes (Fig. 2B, lower panel).

These results were corroborated by in situ hybridization of *CALM1*, *CALM2*, and *CALM3* in adult human hippocampus. We stained hippocampal tissue from two healthy donors using specific RNAscope probes directed at *CALM1*, *CALM2*, or *CALM3* mRNAs (Fig. 2C, Fig S5). Consistent with the GTEx expression results presented above, we observed that all three *CALM* genes were highly expressed across the hippocampal tissue, including in pyramidal cells in the CA regions (Fig. 2C, a1-b5) and in granule cells in the dentate gyrus (Fig. 2C, c1-5).

Together, all three *CALM* genes were consistently highly expressed in the brain, across all examined regions, particularly in neurons. Therefore, although *CALM3* stood out in the statistical risk estimations, we included variants from all three *CALM* genes in subsequent in vitro studies.

### SCZ calmodulin variants affect calcium binding affinity

**Fig. 3:**
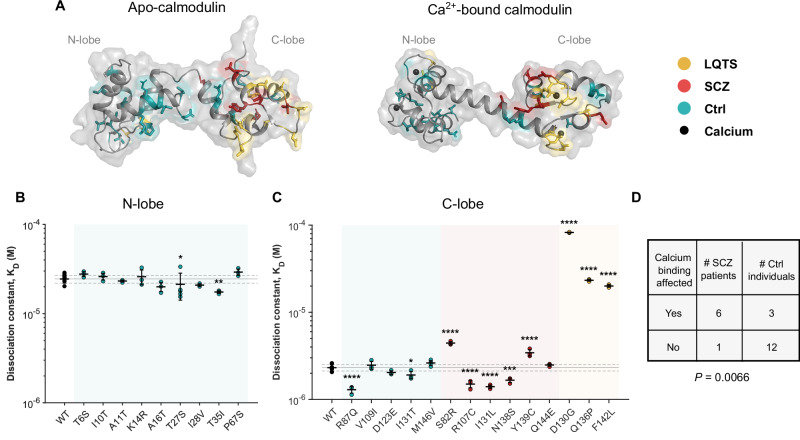
Calmodulin SCZ variants significantly affect calcium binding. **A** Visualization of calmodulin amino acid positions affected in LQTS patients, SCZ patients, and in control individuals on the three-dimensional structure of calmodulin (gray) in apo-state (PDB: 1DMO) or with calcium ions (black) bound (PDB: 1CLL). **B** Calcium affinities of the indicated N-lobe calmodulin variants. **C** Calcium affinities of the indicated C-lobe calmodulin variants. Data in B and C are fitted from binding curves in Fig. S6 and detailed in Table S3-4, n = 3-11 individual experiments. *P < 0.05, ** *P* < 0.01, *** *P* < 0.001, **** *P* < 0.0001 with one-way ANOVA and Dunnett’s *post hoc* test. **D** Summarized effect on calcium binding in SCHEMA carriers of calmodulin variants. Statistics: Fisher’s exact test. Yellow, LQTS; red, SCZ; teal, controls*. Ctrl, control; LQTS, long QT syndrome; K*_*D*_*, dissociation constant; SCZ, schizophrenia*.

The primary function of calmodulin is to bind calcium ions (Fig. 3A). Therefore, we next asked if variants in SCZ patients and control individuals affected the calcium binding affinity of calmodulin. The seven SCZ patients carried six unique variants, as the R107C variant was observed in two individuals. Similarly, in the control group, there were two carriers of I28V, thus 19 unique variants in 20 carriers. We included all six variants from seven SCZ patients, and from the control group all five variants in the C-lobe and nine unique protein variants from ten carriers in the N-lobe. We investigated if the variants affected the calcium binding affinity of the lobe they are located in.

To determine the calcium affinity of each variant, we used a well-established assay in which calcium binding is monitored by the intrinsic phenylalanine (N-lobe) or tyrosine (C-lobe) fluorescence of the protein (Fig S6A-C) [55–57]. From a generic Hill fit, the calcium binding affinity can be determined, represented by the dissociation constant, K_D_ (Fig. 3B, C, Table S3,S4). An increase in the K_D_ value reflects decreased binding affinity. For comparison, we included three LQTS-causing variants with known loss-of-function effects on calmodulin’s calcium affinity: D130G, Q136P, and F142L [12, 19] (Fig. 3C). These arrhythmogenic variants reduced the C-lobe calcium affinity by 8-35-fold compared to the WT. In comparison, all SCZ and control variants changed the calcium affinity by less than 2-fold, regardless of lobe position (Fig. 3B-C, Table S3,S4).

Interestingly, we found significant differences in calcium binding affinity for calmodulin variants in both the SCZ and control group (Fig. 3B, C). Five of the six variants from SCZ patients significantly affected the calcium affinity: S82R and Y139C reduced the calcium binding affinity by 1.9- and 1.5-fold, respectively, compared to the WT – a pattern similar to that of arrhythmogenic variants, albeit with lower magnitude. In contrast, and surprisingly, R107C, I131L, and N138S increased the calcium binding affinity by 1.4-1.5-fold (Fig. 3C, Table S4). Similarly, four variants from the control group, T27S, T35I, R87Q and I131T, increased the affinity by 1.2-1.8-fold, compared to the WT (Fig. 3B, C, Table S3-4). Notably, an increased calcium affinity, a gain-of-function effect, has never before been reported for any human calmodulin variant.

To understand if variants with calcium binding effects were differently distributed between SCZ patients and control individuals, we quantified the number of carriers with significantly altered calcium binding (Fig. 3D). We found that this simple measure – if a variant significantly affects calmodulin calcium sensing – was enough to statistically separate the two groups. Thus, calmodulin variants in SCZ patients were more likely to affect calmodulin calcium sensing than control variants, suggesting a link between the effect of the variant on protein function and SCZ.

### SCHEMA calmodulin variants perturb the apo-structure of calmodulin

Since we found a significant enrichment of calcium binding effects in SCZ patients compared to control individuals, we asked whether the changes in calcium affinity reflected a variant-induced structural change in calmodulin. We noted that among all observed calmodulin variants, the variants from SCZ patients were exclusively in the C-lobe. This remarkable separation (0 SCZ and 15 control variants in N-lobe; 7 SCZ and 5 control variants in C-lobe) was statistically significant (*P* = 0.0009, Fisher’s Exact test). We therefore focused on variants in the C-lobe, including all SCZ variants, all C-lobe control variants, and the three LQTS variants D130G, Q136P, and F142L.

**Fig. 4:**
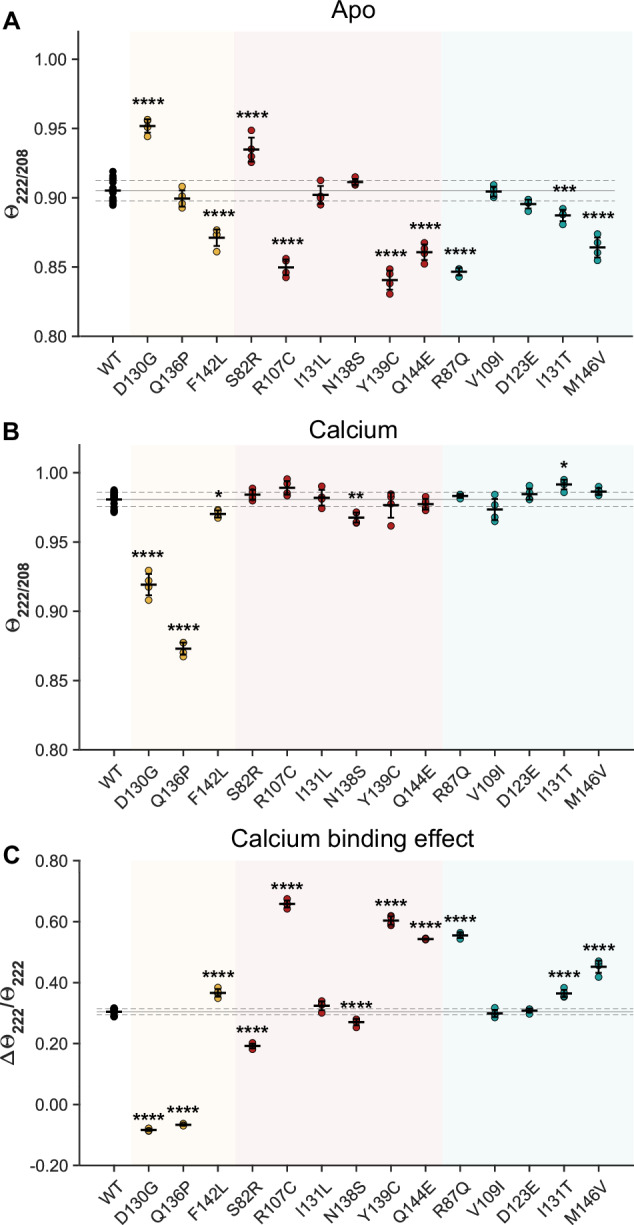
Calmodulin variants in SCHEMA primarily affect the apo structure. Circular dichroism was used to determine structural effects of the indicated variants on the α-helices of apo-calmodulin (**A**) or calcium-bound calmodulin (**B**) as well as the calcium-induced difference (**C**) between the two states (n = 4-16 individual experiments). Data for D130G and Q136P is reproduced from a previous study [63]. *P < 0.05, ** *P* < 0.01, *** *P* < 0.001, **** *P* < 0.0001 with one-way ANOVA with Dunnett’s *post hoc* test. Raw spectra are shown in Fig. S7-8, and summarizing statistics are in Table S5. Yellow, LQTS; red, SCZ; teal, controls. *LQTS: long QT syndrome. SCZ: schizophrenia*.

To explore the structural effects of these variants, we recorded circular dichroism spectra both in the apo-state (Fig S7A-D) and the calcium-bound state (Fig S8A-D). Since calmodulin primarily consists of α-helices in both its forms, we extracted the signal intensity at 222 and 208 nm and calculated the intensity ratio 222/208; a spectral descriptor that reflects the α-helical conformation and environment [58, 59] (Fig. 4A, B, Table S5). To describe the change in α-helical conformation between the apo and calcium-bound state, we determined the change in signal intensity at 222 nm between the two conditions (Fig. 4C, Table S5).

Among the three LQTS-variants, we found that D130G and F142L, but not Q136P, significantly affected the structure of apo-calmodulin (Fig. 4A). On the other hand, D130G and Q136P, but not F142L, strongly affected the structure of calcium-calmodulin (Fig. 4B). Thus, although the calcium affinity was impaired in all three cases, stand-alone structural changes did not necessarily reflect this change and suggested different underlying biophysical mechanisms, likely reflecting the different roles for the affected residues, e.g. if they directly coordinate calcium or not.

For the SCHEMA variants, we found that SCZ variants S82R, R107C, Y139C, and Q144E, and control variants R87Q, I131T, and M146V, had significant structural effects on the apo-form (Fig. 4A). In the calcium-bound form, only SCZ variant N138S and control variant I131T showed significant effects (Fig. 4B). Noteworthy, while the structural effects of the SCZ and control variants were much smaller than the effects of the LQTS variants on the calcium-bound form, they were similar in magnitude to those of the LQTS variants in the apo-form. Considering the change in α-helical content between apo and calcium-bound form, we found that the SCZ variants S82R, R107C, N138S, Y139C, and Q144E and the control variants R87Q, I131T, and M146V had significant effects (Fig. 4C).

Overall, these structural data demonstrated that while LQTS-variants affected both apo- and calcium-bound calmodulin, the variants found in both SCZ patients and control individuals primarily affected apo-calmodulin.

### SCZ calmodulin variants perturb calcium sensitivity during Ca_V_1.2-IQ binding

**Fig. 5:**
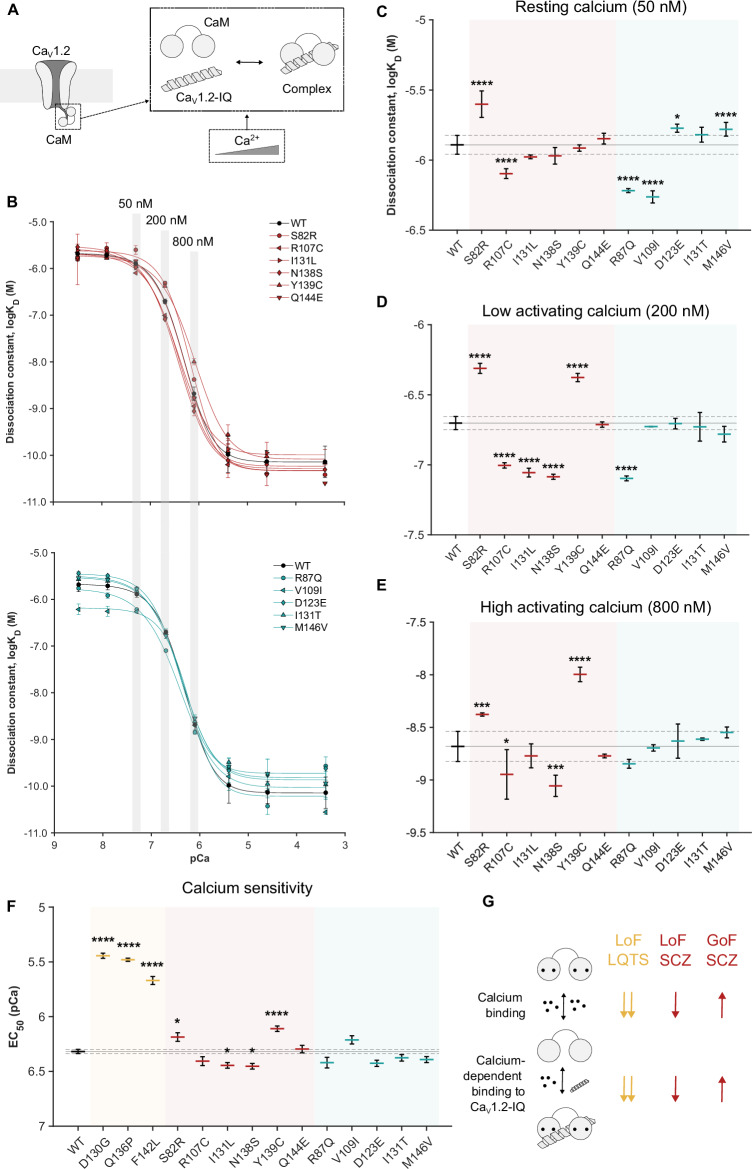
Calmodulin SCZ and control variants have different effects on calcium-dependent Ca_V_1.2-IQ binding. **A** Schematic of calcium-dependent calmodulin (CaM) binding to the IQ-domain of Ca_V_1.2 (Ca_V_1.2-IQ). **B** Binding affinity, represented by the dissociation constant, K_D_, of calmodulin binding to Ca_V_1.2-IQ as a function of free calcium concentration. Vertical gray shades indicate data presented in detail in C-E. **C-E** Calmodulin:Ca_V_1.2-IQ affinity at 50 nM, 200 nM, and 800 nM free calcium, representing three different physiological conditions. **F** EC_50_ values reflecting the calcium sensitivity of calmodulin when bound to the Ca_V_1.2-IQ domain. Data for D130G, Q136P, and F142L is reproduced from previous studies [62, 63]. n = 3-10 individual experiments for B-F, *P < 0.05, *** *P* < 0.001, **** *P* < 0.0001 with one-way ANOVA followed by Dunnett’s *post hoc* test. Statistical calculations for B-F are detailed in Table S6-7, and raw data is shown in Fig S9-11. **G** Schematic summary of calmodulin variant effects on calcium binding and calcium-dependent Ca_V_1.2-IQ binding. Yellow, LQTS; red, SCZ; teal, controls. *EC*_*50*_*, half maximal effective concentration. Ca*_*V*_*1.2: voltage-gated calcium channel 1.2. GoF: gain-of-function. LoF: loss-of-function. LQTS: long QT syndrome. pCa: -log([Ca*^*2+*^*]). SCZ: schizophrenia*.

The pathology of calmodulin variants in LQTS is largely linked to dysregulation of Ca_V_1.2 [3, 16, 18]. Since Ca_V_1.2 is also implicated in SCZ, we asked if calmodulin variants from SCZ patients and control individuals affected Ca_V_1.2 binding and regulation. To answer this question, we determined the dissociation constant, K_D_, for the interaction between calmodulin and the IQ-domain of Ca_V_1.2 (Ca_V_1.2-IQ). The K_D_ value was measured at eight different calcium concentrations spanning resting and activating cellular conditions [45] (Fig. 5A, Fig S9-11). Across this range of calcium concentrations, the binding affinity of calmodulin towards Ca_V_1.2-IQ increases by more than 10,000 fold, emphasizing the sophisticated calcium sensing function of calmodulin (Fig. 5B).

At low resting calcium concentrations (50 nM, Fig. 5C) [60, 61], we found that one SCZ variant (S82R) and two control variants (D123E and M146V) caused a significant decrease in the Ca_V_1.2-IQ affinity of calmodulin (loss-of-function), whereas another SCZ variant (R107C) and two other control variants (R87Q and V109I) caused a significant increase in affinity (gain-of-function). Initially in the calcium-induced transition from low to high Ca_V_1.2-IQ affinity (200 nM free calcium, Fig. 5D), we found that all SCZ variants, except Q144E, significantly changed the Ca_V_1.2-IQ affinity, albeit in different directions: S82R and Y139C decreased the affinity, whereas R107C, I131L, and N138S increased it. In the control group, one variant (R87Q) significantly increased the affinity. Later in the calcium-induced transition (800 nM free calcium, Fig. 5E), four SCZ variants retained their significant effects on the Ca_V_1.2-IQ interaction (S82R, R107C, N138S, and Y139C), whereas the control group variants no longer had any significant effect.

Finally, at high calcium concentrations ( > 800 nM) reflecting a calcium-saturated calmodulin:Ca_V_1.2-IQ complex, the affinity was too high to determine accurately, and statistical testing could thus only be done for a subset of the calmodulin variants. For those measured, there were no significant effects at these calcium concentrations (Table S6).

As an overall measure of the variant effect on the calcium-dependency of calmodulin binding to Ca_V_1.2-IQ, we determined the EC_50_ value for all calmodulin:Ca_V_1.2-IQ affinity curves (Fig. 5F, Table S7). For comparison, we included already published data for the three LQTS variants D130G, Q136P, and F142L [62, 63]. Among the calmodulin variants from SCZ patients, we found that S82R and Y139C significantly increased the EC_50_ value, suggesting a reduction in the calcium sensitivity of calmodulin in the calmodulin:Ca_V_1.2-IQ complex. On the contrary, SCZ variants I131L and N138S significantly decreased the EC_50_ value, suggesting an increased calcium sensitivity of calmodulin in the complex. Remarkably, among the calmodulin variants from control individuals, we found no statistical differences from the WT. Of note, compared to the EC_50_ effect of the three calmodulin variants in LQTS patients, the effects in SCZ patients were substantially smaller in magnitude.

The effects of the SCZ calmodulin variants on Ca_V_1.2-IQ binding affinity and calcium sensitivity correlated excellently with the effects on calcium binding (Fig. 5G). S82R and Y139C, found to reduce the calcium affinity of calmodulin, impaired Ca_V_1.2-IQ binding and calcium sensitivity. In contrast, R107C, I131L, and N138S, found to increase the calcium affinity of calmodulin, enhanced Ca_V_1.2-IQ binding and calcium sensitivity.

### The SCZ calmodulin variant Y139C impairs Ca_V_1.2 closure

**Fig. 6:**
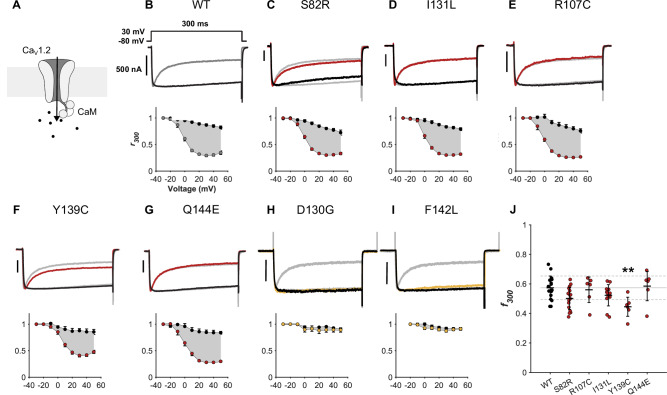
Small effects of calmodulin variants from SCZ patients on CDI of the Ca_V_1.2 channel. **A** Calmodulin binds the C-terminal tail of Ca_V_1.2 and regulates channel closure in a process termed calcium dependent inactivation (CDI). **B-I** Top panels show exemplar currents evoked by 30-mV voltage step in cells co-transfected with Ca_V_1.2 and the indicated calmodulin variants. CDI manifests as the rapid decay in calcium current (colored trace) as compared to barium (black trace). For comparison, the trace for WT calmodulin is reproduced in gray behind the exemplar traces for the indicated variants. Bottom panels show population data for CDI across voltages. *r*_*300*_ measures the current remaining after 300 ms, after normalization to peak current. Data in H and I is reproduced from a previous study along with the corresponding WT in gray [18]. **J** Quantification of *f*_*300*_, the difference between calcium and barium at 30 mV, after normalization by the barium *r*_300_ value. n = 6-16 individual experiments, ** *P* < 0.01 with one-way ANOVA followed by Dunnett’s *post hoc* test. Statistical calculations are detailed in Table S8. *Ca*_*V*_*1.2: voltage-gated calcium channel 1.2. SCZ: schizophrenia*.

Calmodulin binding to Ca_V_1.2 and subsequent calcium sensing, is translated into CDI of the channel (Fig. 6A). Since calmodulin variants from SCZ patients, but not control individuals, affected the calcium-sensitivity of calmodulin when bound to Ca_V_1.2-IQ, we asked if these differences translated to an effect on Ca_V_1.2 CDI, using whole-cell voltage-clamp recordings of transfected HEK293 cells.

Cells were clamped at defined voltages (Fig. 6B-I). Upon clamping, the channels open, and the inward ion current is visualized as a downward deflection of the exemplar current traces (Fig. 6B-I, top panels). Shortly after, a rapid decay in calcium current (red or yellow trace) is inflicted by calmodulin in response to sensing calcium, thereby resulting in CDI. For reference, the same experiment was conducted with barium instead of calcium. Calmodulin cannot bind barium ions and thus does not induce CDI, and the barium current remains at almost the same level during the experiment (black trace). The calcium and barium currents remaining after 300 ms were quantified as *r*_*300*_, and the difference between the two was calculated as *f*_*300*_ (Fig. 6B-I, lower panels, Table S8, Fig S12).

None of the SCZ variants (Fig. 6B-G) affected CDI to similar levels as the LQTS variants D130G and F142L (Fig. 6H-I). The relatively modest effects could not be attributed to lower expression levels, as all calmodulin variants were overexpressed compared to endogenous levels, and all but the Q144E variant overexpressed to higher levels than WT (Fig S1B). Among the SCZ variants, a significant reduction of 24.8% in *f*_*300*_ was observed for Y139C (0.57 ± 0.08 to 0.445 ± 0.06, Fig. 6F,J) consistent with a gain-of-function effect on the channel. Although not reaching statistical significance, a small reduction was also observed for S82R (12.5% reduction to 0.50 ± 0.08, Fig. 6C,J). Interestingly, S82R and Y139C were the only SCZ variants that reduced the calcium affinity of calmodulin and the calcium sensing of calmodulin in the Ca_V_1.2-IQ:calmodulin complex, suggesting that they act through a similar mechanism to that of LQTS patients, but with a smaller impact. Together, these data demonstrated that calmodulin variants from SCZ patients had small or no effects on Ca_V_1.2 CDI.

## Discussion

Our framework for understanding the effects of human calmodulin variants comes from cardiac arrhythmia, primarily LQTS. LQTS variants generally have strong loss-of-function effects on C-lobe calcium affinity and strongly impair Ca_V_1.2 regulation. Of note, the loss-of-function effects of calcium binding to calmodulin translate to gain-of-function effects on the channel, as channel closure is compromised and calcium entry is increased [3, 18]. Calmodulin is constitutively bound to Ca_V_1.2 and remains associated with the channel in both resting and activating conditions [22, 64]. Thus, a pathogenic calmodulin variant that binds Ca_V_1.2 equally well as the WT will occupy some of the Ca_V_1.2 binding sites without being able to properly respond to calcium signals to facilitate CDI. Consequently, even though patients only carry a variant in one in six alleles, it can cause a dominant phenotype.

Similar to cardiac arrhythmia patients, all SCZ variants identified in this study occur in the calmodulin C-lobe. These observations suggest that the C-lobe is particularly sensitive to genetic alterations. Pre-association of calmodulin with Ca_V_1.2 takes place through the C-lobe. In this position, the C-lobe can rapidly respond to calcium changes to regulate CDI. Thus, changes in C-lobe calcium affinity perturb its calcium sensing function and impair CDI [16, 18, 46]. Similar C-lobe-mediated pre-association is observed for a number of other calmodulin targets that are expressed in cardiac and neuronal cells such as the ryanodine receptors [45] and the voltage-gated sodium channels [65]. We suggest that this indispensable calcium sensing role of the calmodulin C-lobe explains why C-lobe variants are particularly linked to disease.

Despite the shared location to the C-lobe, there was no overlap in amino acid substitutions between SCZ variants and cardiac variants, and only a single positional overlap on N138 [13]. This difference translated into different molecular effects of the two groups of variants: the cardiac LQTS variants consistently and strongly impaired calcium affinity, altered protein structure, and impaired Ca_V_1.2 binding and regulation. The SCZ variants had differential effects with smaller effect size. The S82R and Y139C variants impaired calcium and Ca_V_1.2-IQ binding. Y139C also impacted Ca_V_1.2 closure, whereas S82R had a small effect that did not reach statistical significance. Contrary to this, R107C, I131L, and N138S potentiated calcium and Ca_V_1.2-IQ binding affinity, but had no effects on Ca_V_1.2 regulation in our assay. We propose that these differences in effect size determine whether a carrier suffers from LQTS or not.

It was surprising that three of the variants had gain-of-function effects on calcium and Ca_V_1.2-IQ binding affinity. To our knowledge, such gain-of-function effects have not previously been reported for a human calmodulin variant. As these effects did not translate to an overt effect on Ca_V_1.2 regulation, we speculate if the variants could affect other brain targets through an alternative mechanistic paradigm. Alternative pathways may involve other ion channels or enzymes where calmodulin binding and regulation has a different calcium dependency. CaMKII is activated by calcium-bound calmodulin as an on/off switch that feeds forward and activates numerous cellular signaling cascades. Here, even a small gain-of-function effect on calmodulin calcium binding may be amplified to cause a strong cellular impact.

Although the effects of calmodulin SCZ variants were smaller than LQTS variant effects, they were consistently larger and/or more frequent than those of the control variants, suggesting different molecular effects of the SCZ and control group. Despite the designation ‘control group’, the observation of significant effects among this group of calmodulin variants was not unexpected. First, the calmodulin protein sequence is highly conserved, suggesting a loss-of-fitness from any kind of variation [8]. Second, the control population enrolled in the SCHEMA study is a combination of individuals with no known psychiatric disease, individuals from other non-neurological populations, and individuals randomly selected from population registries [40]. Thus, not all controls are directly screened for SCZ, and none are systematically screened for heart disease or other conditions. In principle, these individuals could therefore be at risk of having or developing a psychiatric disorder or other diseases. We do, however, not expect such other phenotypes to be highly detrimental, as that would likely have prevented participation in a genetic screening study as adults. We included calmodulin variants from both SCZ cases and control individuals in the SCHEMA cohort to capture the full allelic spectrum of variants and their functional consequences. Moreover, having such unscreened controls is customary in most genetic association studies.

This study identified calmodulin variants in SCZ patients, complementing reports of intellectual disability, seizures, ADHD, and ASD in patients in the ICalmR. This broad range of neurological conditions is not surprising, as genetic studies suggest a large overlap in the genetic factors underlying neurodevelopmental disorders, including ADHD, ASD, intellectual disability, and SCZ [66, 67]. SCZ, as well as other neurodevelopmental disorders, are highly heritable and large sequencing studies – including SCHEMA – have identified long lists of genetic variants contributing to these diseases. Together, they paint a complex picture of rare genetic variants associated with relatively high disease risk as well as many common genetic variants associated with an individual small disease risk. In SCZ, genetic variants include deletions of large chromosomal regions, protein-truncating variants, small insertions, and deletions, as well as missense variants. Thus, SCZ is a highly polygenic disease, and the current understanding is that several genetic factors act together to contribute to the development of SCZ in most cases. This may also apply to variants in the calmodulin genes. In cardiac patients, such variants typically have strong functional effects and act in a monogenic manner to cause disease. In contrast, calmodulin variants with more modest functional consequences may contribute to SCZ through polygenic mechanisms, acting in concert with numerous other genetic variants and potentially involving multiple molecular pathways.

In conclusion, we identified calmodulin variants in SCZ patients. Our mechanistic findings suggested that these variants act through two different mechanistic paradigms. In one paradigm, SCZ variants impaired calcium affinity and Ca_V_1.2 binding and regulation similarly to variants from LQTS patients, but with a much smaller effect size. In an alternative paradigm, SCZ variants enhanced calcium affinity and Ca_V_1.2 binding but had no discernible functional impact on Ca_V_1.2. Therefore, we propose that both paradigms, but particularly the latter, act through other or additional molecular targets in the brain to those known from LQTS.

## Supplementary information

Supplementary information

## Data Availability

A large portion of the raw data is presented in the Supplementary Information. All data is available upon request.
