# Supplementary material for: Functional consequences of calmodulin variants identified among schizophrenia patients and controls
Source: Transl Psychiatry. 2025 Nov 22;15:529. doi: 10.1038/s41398-025-03735-3 (PMC12711917; doi:10.1038/s41398-025-03735-3)
Supplement: Supplementary file 1 — Supplementary information [file 41398_2025_3735_MOESM1_ESM.docx]

Supplementary Information for

Functional consequences of calmodulin variants identified among schizophrenia patients and controls

Helene Halkjær Jensen, PhD^1,^*, Malene Brohus, PhD^1,^*, John W. Hussey III, PhD^2^, Ana-Octavia Busuioc, MSc^1^, Emil Drivsholm Iversen, MSc^1^, Faezeh Darki, MSc^3^, Gabriela Dobromirova Nikolova, MSc^1^, Amalie Elton Baisgaard, MSc^3^, Palle Duun Rohde, PhD^3^, Ida Elisabeth Gad Holm, M.D.^4^, Andrew McQuillin, PhD^5^, Anders Olsen, PhD^1^, Torben Moos, PhD^3^, Ivy E. Dick, PhD^2^, Michael Toft Overgaard, PhD^1,$^, Mette Nyegaard PhD^3,6 $^

^1^Department of Chemistry and Bioscience, Aalborg University, Aalborg, Denmark

^2^Department of Pharmacology and Physiology, University of Maryland School of Medicine, Baltimore, MD, USA

^3^Department of Health Science and Technology, Aalborg University, Aalborg, Denmark

^4^Department of Pathology, Aalborg University Hospital, Aalborg, Denmark

^5^Division of Psychiatry, University College London, London, United Kingdom

^6^ Statens Serum Institut, Copenhagen, Denmark

* Authors contributed equally, ^$^ Authors contributed equally

**Supplementary tables**

| **Gene** | **Genomic position (hg19)** | **Nucleotide change** | **Variant** | **Variant** | **Protein variant** | **Protein variant** | **Phenotype** | **Count** | **Lobe** | **MPC score** | **CADD score** |
| --- | --- | --- | --- | --- | --- | --- | --- | --- | --- | --- | --- |
| *CALM1* | 14-90867614-G-A | c.46G>A | p.Ala16Thr | A16T | Ala15Thr | A15T | Control | 1 | N | 2.68 | 26.8 |
|  | 14-90870226-C-T | c.199C>T | p.Pro67Ser | P67S | Pro66Ser | P66S | Control | 1 | N | 1.88 | 23.2 |
|  | 14-90870273-T-G | c.246T>G | p.Ser82Arg | S82R | Ser81Arg | S81R | SCZ case | 1 | C | 2.03 | 17.0 |
|  | 14-90870756-C-T | c.319C>T | p.Arg107Cys | R107C | Arg106Cys | R106C | SCZ case | 1 | C | 2.24 | 24.3 |
|  | 14-90870762-G-A | c.325G>A | p.Val109Ile | V109C | Val108Cys | V108C | Control | 1 | C | 1.66 | 22.5 |
|  | 14-90871041-C-G | c.430C>G | p.Gln144Glu | Q144E | Gln143Glu | Q143E | SZ case | 1 | C | 1.83 | 18.8 |
|  |  |  |  |  |  |  |  |  |  |  |  |
| *CALM2* | 2-47397890-G-C | c.17C>G | p.Thr6Ser | T6S | Thr5Ser | T5S | Control | 1 | N | 1.50 | 22.5 |
|  | 2-47389795-T-C | c.41A>G | p.Lys14Arg | K14R | Lys13Arg | K13R | Control | 1 | N | 1.53 | 18.2 |
|  | 2-47389756-G-C | c.80C>G | p.Thr27Ser | T27S | Thr26Ser | T26S | Control | 1 | N | 1.51 | 17.4 |
|  | 2-47389732-G-A | c.104C>T | p.Thr35Ile | T35I | Thr34Ile | T34I | Control | 1 | N | 2.37 | 24.7 |
|  | 2-47388964-G-A | c.319C>T | p.Arg107Cys | R107C | Arg106Cys | R106C | SCZ case | 1 | C | 2.13 | 24.0 |
|  | 2-47388892-T-G | c.391A>C | p.Ile131Leu | I131L | Ile130Leu | I130L | SCZ case | 1 | C | 1.63 | 15.5 |
|  | 2-47388891-A-G | c.392T>C | p.Ile131Thr | I131T | Ile130Thr | I130T | Control | 1 | C | 1.98 | 14.0 |
|  | 2-47388867-T-C | c.416A>G | p.Tyr139Cys | Y139C | Tyr138Cys | Y138C | SCZ case | 1 | C | 2.80 | 25.8 |
|  |  |  |  |  |  |  |  |  |  |  |  |
| *CALM3* | 19-47109087-G-A | c.7G>A | p.Asp3Asn | D3N | Asp2Asn | D2N | Control | 1 | N | 1.51 | 23.5 |
|  | 19-47109109-T-C | c.29T>C | p.Ile10Thr | I10T | Ile9Thr | I9T | Control | 1 | N | 1.94 | 23.6 |
|  | 19-47109111-G-A | c.31G>A | p.Ala11Thr | A11T | Ala10Thr | A10T | Control | 1 | N | 1.52 | 23.5 |
|  | 19-47111454-A-T | c.35A>T | p.Glu12Val | E12V | Glu11Val | E11V | Control | 1 | N | 2.74 | 28.9 |
|  | 19-47111501-A-G | c.82A>G | p.Ile28Val | I28V | Ile27Val | I27V | Control | 2 | N | 1.65 | 23.6 |
|  | 19-47111505-C-G | c.86C>G | p.Thr29Ser | T29S | Thr28Ser | T28S | Control | 1 | N | 1.92 | 22.2 |
|  | 19-47111559-C-G | c.140C>G | p.Ala47Gly | A47G | Ala46Gly | A46G | Control | 1 | N | 1.54 | 23.3 |
|  | 19-47111744-G-A | c.184G>A | p.Gly62Arg | G62R | Gly61Arg | G61R | Control | 1 | N | 3.02 | 34.0 |
|  | 19-47111820-G-A | c.260G>A | p.Arg87Gln | R87Q | Arg86Gln | R86Q | Control | 1 | C | 1.76 | 24.8 |
|  | 19-47112186-T-G | c.369T>G | p.Asp123Glu | D123E | Asp122Glu | D122E | Control | 1 | C | 1.45 | 14.7 |
|  | 19-47112230-A-G | c.413A>G | p.Asn138Ser | N138S | Asn137Ser | N137S | SCZ case | 1 | C | 1.30 | 21.7 |
|  | 19-47112396-A-G | c.436A>G | p.Met146Val | M146V | Met145Val | M145V | Control | 1 | C | 1.60 | 21.1 |

**Table S1: *CALM1-3* missense variants identified in SCHEMA.** Missense variants in *CALM1-3* were extracted from the SCHEMA browser. MPC and CADD pathogenicity prediction scores were also extracted from the SCHEMA browser.

|  | **SCZ**  ***CALM* carrier**  (n = 24248) | **Controls**  ***CALM* carrier**  (n = 97322) | **OR** | **95% CI lower** | **95% CI upper** | ***P*-value** | ***P_adj_*-value** |
| --- | --- | --- | --- | --- | --- | --- | --- |
| ***CALM1+2+3*** | 4 | 4 | 4.01 | 0.95 | 17 | 0.058 | 0.14 |
| N-lobe | 0 | 4 | 2.80 E-04 | NA | 0.51 | 0.18 | 0.28 |
| C-lobe | 4 | 0 | 5.70 E+04 | 31.73 | NA | 0.00033 | 0.0036 |
| ***CALM1*** | 2 | 1 | 8.03 | 0.77 | 0.17 E+03 | 0.079 | 0.14 |
| N-lobe | 0 | 1 | 7.60 E-04 | NA | 0.15 E+03 | 0.5 | 0.5 |
| C-lobe | 2 | 0 | 5.70 E+04 | 1.2 | NA | 0.011 | 0.04 |
| ***CALM2*** | 2 | 1 | 8.03 | 0.77 | 0.17 E+03 | 0.079 | 0.14 |
| N-lobe | 0 | 1 | 7.60 E-04 | NA | 0.15 E+03 | 0.5 | 0.5 |
| C-lobe | 2 | 0 | 5.70 E+04 | 1.2 | NA | 0.011 | 0.04 |
| ***CALM3*** | 0 | 2 | 2.80 E-04 | NA | 14 | 0.34 | 0.42 |
| N-lobe | 0 | 2 | 2.80 E-04 | NA | 14 | 0.34 | 0.42 |
| C-lobe | 0 | 0 | NA | NA | NA | NA | NA |

**Table S2: Odds ratio calculations for calmodulin variants with MPC ≥2.** The MPC score for all calmodulin variants in SCHEMA were extracted in the browser (Table S1). The MPC score integrates estimates of the deleteriousness of a particular amino acid substitution in constrained genes, PolyPhen-2 predictions of functional effects of a particular amino acid substitution, and how constrained the gene is. In SCHEMA, ≥2 is considered a high pathogenicity score. Here, we calculated SCZ risk for calmodulin variants with MPC ≥2. P_adj_-values are false discovery rate (FDR) corrected.

| **Protein variant** | **Carrier phenotype** | **Apparent N-lobe**  **dissociation constant,**  ***K*_D,app_(μM)** | | **n** | **Fold change  from WT** | ***P*-value** | **Summary** |
| --- | --- | --- | --- | --- | --- | --- | --- |
|  |  | **Mean** | **SD** |  |  |  |  |
| **WT** |  | 2.45 E-05 | 2.51 E-06 | 11 | 1.00 |  |  |
| **T6S** | Ctrl | 2.77 E-05 | 1.73 E-06 | 3 | 1.13 | 0.95 | ns |
| **I10T** | Ctrl | 2.60 E-05 | 2.27 E-06 | 3 | 1.06 | 1.0 | ns |
| **A11T** | Ctrl | 2.32 E-05 | 6.51 E-07 | 3 | 0.95 | 0.98 | ns |
| **K14R** | Ctrl | 2.59 E-05 | 4.94 E-06 | 3 | 1.06 | 1.0 | ns |
| **A16T** | Ctrl | 2.00 E-05 | 2.27 E-06 | 3 | 0.82 | 0.14 | ns |
| **T27S** | Ctrl | 2.13 E-05 | 7.15 E-06 | 6 | 0.87 | 0.011 | * |
| **I28V** | Ctrl | 2.09 E-05 | 7.08 E-07 | 3 | 0.85 | 0.37 | ns |
| **T35I** | Ctrl | 1.75 E-05 | 5.90 E-07 | 4 | 0.71 | 0.0014 | ** |
| **P67S** | Ctrl | 2.92 E-05 | 2.39 E-06 | 3 | 1.20 | 0.67 | ns |

**Table S3: Calcium binding affinity to N-lobe SCHEMA variants.** Calcium affinities of the calmodulin N-lobe, represented by the apparent dissociation constant, K_D,app_. Fold changes were calculated for each calmodulin variant by division with the WT affinity. *P*-values were determined using one-way ANOVA with Dunnett’s multiple comparisons test. *SD, standard deviation*.

| **Protein variant** | **Carrier phenotype** | **Apparent C-lobe**  **dissociation constant,**  ***K*_D,app_(μM)** | | **n** | **Fold change  from WT** | ***P*-value** | **Summary** |
| --- | --- | --- | --- | --- | --- | --- | --- |
|  |  | **Mean** | **SD** |  |  |  |  |
| **WT** |  | 2.31 E-06 | 1.95 E-07 | 4 |  |  |  |
| **D130G** | LQTS | 8.24 E-05 | 7.55 E-07 | 3 | 36 | < 0.0001 | **** |
| **Q136P** | LQTS | 2.33 E-05 | 5.76 E-07 | 3 | 10 | < 0.0001 | **** |
| **F142L** | LQTS | 2.01 E-05 | 6.74 E-07 | 3 | 8.7 | < 0.0001 | **** |
| **S82R** | SCZ | 4.43 E-06 | 1.74 E-07 | 3 | 1.9 | < 0.0001 | **** |
| **R107C** | SCZ | 1.50 E-06 | 1.40 E-07 | 3 | 0.65 | < 0.0001 | **** |
| **I131L** | SCZ | 1.40 E-06 | 6.24 E-08 | 3 | 0.61 | < 0.0001 | **** |
| **N138S** | SCZ | 1.66 E-06 | 1.01 E-07 | 3 | 0.72 | 0.0001 | *** |
| **Y139C** | SCZ | 3.43 E-06 | 2.88 E-07 | 3 | 1.5 | < 0.0001 | **** |
| **Q144E** | SCZ | 2.49 E-06 | 8.25 E-08 | 3 | 1.1 | 0.89 | ns |
| **R87Q** | Ctrl | 1.29 E-06 | 1.14 E-07 | 3 | 0.56 | < 0.0001 | **** |
| **V109I** | Ctrl | 2.47 E-06 | 2.55 E-07 | 3 | 1.1 | 0.95 | ns |
| **D123E** | Ctrl | 2.04 E-06 | 9.68 E-08 | 3 | 0.88 | 0.35 | ns |
| **I131T** | Ctrl | 1.91 E-06 | 1.85 E-07 | 3 | 0.82 | 0.037 | * |
| **M146V** | Ctrl | 2.62 E-06 | 1.70 E-07 | 4 | 1.1 | 0.26 | ns |

**Table S4: Calcium binding affinity to C-lobe SCHEMA variants.** Calcium affinities of the calmodulin C-lobe, represented by the apparent dissociation constant, K_D,app_. Fold changes were calculated for each calmodulin variant by division with the WT affinity. *P*-values were determined using one-way ANOVA with Dunnett’s *post hoc* test. *SD, standard deviation*.

| **Protein variant** | **Carrier phenotype** | **n** | **Apo calmodulin**  **Θ_222/208_** | | | | **Calcium bound calmodulin**  **Θ_222/208_** | | | | **Apo-calcium relation**  **ΔΘ_222_/Θ_222_** | | | |
| --- | --- | --- | --- | --- | --- | --- | --- | --- | --- | --- | --- | --- | --- | --- |
|  |  |  | **Mean** | **SD** | ***P-value*** | **Summary** | **Mean** | **SD** | ***P-value*** | **Summary** | **Mean** | **SD** | ***P-value*** | **Summary** |
| **WT** |  | 16 | 0.905 | 0.007 |  |  | 0.981 | 0.005 |  |  | 0.304 | 0.01 |  |  |
| **D130G** | LQTS | 4 | 0.952 | 0.005 | < 0.0001 | **** | 0.919 | 0.008 | < 0.0001 | **** | -0.083 | 0.00 | < 0.0001 | **** |
| **Q136P** | LQTS | 4 | 0.899 | 0.006 | 0.67 | ns | 0.873 | 0.004 | < 0.0001 | **** | -0.067 | 0.00 | < 0.0001 | **** |
| **F142L** | LQTS | 4 | 0.871 | 0.006 | < 0.0001 | **** | 0.97 | 0.003 | 0.02 | * | 0.366 | 0.01 | < 0.0001 | **** |
| **S82R** | SCZ | 4 | 0.935 | 0.009 | < 0.0001 | **** | 0.984 | 0.003 | 0.94 | ns | 0.192 | 0.01 | < 0.0001 | **** |
| **R107C** | SCZ | 4 | 0.85 | 0.006 | < 0.0001 | **** | 0.989 | 0.005 | 0.10 | ns | 0.658 | 0.01 | < 0.0001 | **** |
| **I131L** | SCZ | 4 | 0.902 | 0.006 | 0.99 | ns | 0.982 | 0.006 | 1.0 | ns | 0.324 | 0.01 | 0.13 | ns |
| **N138S** | SCZ | 4 | 0.911 | 0.002 | 0.58 | ns | 0.968 | 0.004 | 0.0018 | ** | 0.270 | 0.01 | < 0.0001 | **** |
| **Y139C** | SCZ | 4 | 0.841 | 0.007 | < 0.0001 | **** | 0.977 | 0.009 | 0.84 | ns | 0.604 | 0.01 | < 0.0001 | **** |
| **Q144E** | SCZ | 4 | 0.861 | 0.006 | < 0.0001 | **** | 0.977 | 0.004 | 0.94 | ns | 0.543 | 0.00 | < 0.0001 | **** |
| **R87Q** | Ctrl | 4 | 0.847 | 0.003 | < 0.0001 | **** | 0.977 | 0.011 | 1.0 | ns | 0.555 | 0.01 | < 0.0001 | **** |
| **V109I** | Ctrl | 4 | 0.905 | 0.004 | 1.0 | ns | 0.973 | 0.008 | 0.21 | ns | 0.299 | 0.01 | 0.94 | ns |
| **D123E** | Ctrl | 4 | 0.895 | 0.003 | 0.11 | ns | 0.985 | 0.004 | 0.89 | ns | 0.308 | 0.01 | 1.0 | ns |
| **I131T** | Ctrl | 4 | 0.887 | 0.004 | 0.00020 | *** | 0.991 | 0.003 | 0.016 | * | 0.364 | 0.01 | < 0.0001 | **** |
| **M146V** | Ctrl | 4 | 0.864 | 0.007 | < 0.0001 | **** | 0.986 | 0.002 | 0.50 | ns | 0.452 | 0.02 | < 0.0001 | **** |

**Table S5: Circular dichroism structural effect measures and statistics.** Values at 208 and 222 nm were extracted from circular dichroism spectra (Fig S7 and S8). These numbers were used to calculate the Θ_222/208_ for apo and calcium bound calmodulin as well as ΔΘ_222_/Θ_222_. Statistical differences between WT and SCZ or control variants were determined with a one-way ANOVA followed by Dunnett’s *post hoc* test. *SD, standard deviation*.

| **K_D_ (M)** | | | | | | | | | | | | | | | | | |
| --- | --- | --- | --- | --- | --- | --- | --- | --- | --- | --- | --- | --- | --- | --- | --- | --- | --- |
| **CaM variant** | **Pheno-type** | **Mean** | **SD** | **n** | ***P*** | **Mean** | **SD** | **n** | ***P*** | **Mean** | **SD** | **n** | ***P*** | **Mean** | **SD** | **n** | ***P*** |
| **[Ca^2+^]_free_[M]** | |  | **3.16E-09** | | |  | **1.26E-08** | | |  | **5.01E-08** | | |  | **2.00E-07** | | |
| **WT** |  | 2.18E-06 | 6.80E-07 | 10 | - | 1.97E-06 | 3.65E-07 | 10 | - | 1.30E-06 | 2.06E-07 | 10 | - | 1.99E-07 | 2.15E-08 | 10 | - |
| **S82R** | SCZ | 2.61E-06 | 3.07E-06 | 3 | 8.9E-01 | 2.79E-06 | 6.80E-07 | 3 | **2.6E-02** | 2.54E-06 | 5.45E-07 | 2 | **< 0.0001** | 4.88E-07 | 3.97E-08 | 3 | **< 0.0001** |
| **R107C** | SCZ | 2.23E-06 | 1.80E-07 | 3 | 1.0E+00 | 1.76E-06 | 1.58E-07 | 3 | 9.5E-01 | 8.03E-07 | 6.25E-08 | 3 | **< 0.0001** | 9.91E-08 | 4.44E-09 | 3 | **< 0.0001** |
| **I131L** | SCZ | 2.49E-06 | 4.72E-07 | 3 | 9.9E-01 | 2.17E-06 | 3.09E-07 | 3 | 9.5E-01 | 1.06E-06 | 3.22E-08 | 3 | 1.2E-01 | 8.81E-08 | 6.31E-09 | 3 | **< 0.0001** |
| **N138S** | SCZ | 2.90E-06 | 4.15E-07 | 3 | 7.7E-01 | 2.43E-06 | 1.53E-07 | 3 | 2.7E-01 | 1.08E-06 | 1.48E-07 | 3 | 1.8E-01 | 8.22E-08 | 3.52E-09 | 3 | **< 0.0001** |
| **Y139C** | SCZ | 1.77E-06 | 2.05E-07 | 3 | 1.0E+00 | 1.69E-06 | 1.15E-07 | 3 | 7.5E-01 | 1.22E-06 | 6.30E-08 | 3 | 9.9E-01 | 4.20E-07 | 2.86E-08 | 3 | **< 0.0001** |
| **Q144E** | SCZ | 1.69E-06 | 1.77E-07 | 3 | 9.8E-01 | 1.92E-06 | 2.27E-07 | 3 | 1.0E+00 | 1.43E-06 | 1.23E-07 | 3 | 7.7E-01 | 1.94E-07 | 8.51E-09 | 3 | 1.0E+00 |
| **R87Q** | Ctrl | 1.73E-06 | 9.86E-08 | 3 | 9.9E-01 | 1.21E-06 | 1.64E-07 | 3 | **1.0E-03** | 6.06E-07 | 2.05E-08 | 3 | **< 0.0001** | 7.99E-08 | 3.24E-09 | 3 | **< 0.0001** |
| **V109I** | Ctrl | 6.25E-07 | 1.75E-07 | 3 | **3.0E-04** | 5.67E-07 | 1.40E-07 | 3 | **< 0.0001** | 5.48E-07 | 5.36E-08 | 3 | **< 0.0001** | 1.87E-07 | 5.69E-10 | 3 | 9.7E-01 |
| **D123E** | Ctrl | 3.63E-06 | 2.77E-07 | 3 | 2.1E-01 | 3.21E-06 | 2.27E-07 | 3 | **5.0E-04** | 1.69E-06 | 1.12E-07 | 3 | **1.3E-02** | 1.97E-07 | 1.71E-08 | 3 | 1.0E+00 |
| **I131T** | Ctrl | 2.71E-06 | 1.52E-07 | 3 | 9.1E-01 | 2.48E-06 | 3.69E-07 | 3 | 2.2E-01 | 1.52E-06 | 1.91E-07 | 3 | 2.6E-01 | 1.90E-07 | 4.65E-08 | 3 | 9.5E-01 |
| **M146V** | Ctrl | 2.89E-06 | 3.94E-07 | 3 | 7.7E-01 | 2.64E-06 | 2.39E-07 | 3 | 5.8E-02 | 1.67E-06 | 1.93E-07 | 3 | **2.3E-02** | 1.66E-07 | 2.06E-08 | 3 | 7.1E-02 |
| **[Ca^2+^]_free_[M]** | |  | **7.94E-07** | | |  | **3.98E-06** | | |  | **2.51E-05** | | |  | **3.98E-04** | | |
| **WT** |  | 2.19E-09 | 6.88E-10 | 10 | - | 1.45E-10 | 1.23E-10 | 8 | - | 8.29E-11 | 5.07E-11 | 4 | - | 9.34E-11 | 7.20E-11 | 7 | - |
| **S82R** | SCZ | 4.19E-09 | 1.47E-10 | 3 | **3.5E-03** | 1.28E-10 | 6.01E-11 | 3 | 1.0E+00 | 4.33E-11 | 2.35E-11 | 2 | 8.0E-01 | 3.84E-11 | 9.55E-13 | 2 | 6.9E-01 |
| **R107C** | SCZ | 1.25E-09 | 6.93E-10 | 3 | **1.2E-02** | 7.53E-11 | 5.83E-11 | 2 | 9.6E-01 | 9.62E-11 | 8.64E-11 | 3 | 1.0E+00 | NA | NA | 0 | NA |
| **I131L** | SCZ | 1.74E-09 | 4.84E-10 | 3 | 8.5E-01 | 1.47E-10 | 7.01E-11 | 3 | 1.0E+00 | 1.24E-10 | 6.34E-11 | 3 | 9.0E-01 | NA | NA | 0 | NA |
| **N138S** | SCZ | 8.95E-10 | 2.00E-10 | 3 | **3.0E-04** | 9.66E-11 | 7.37E-11 | 3 | 1.0E+00 | 6.40E-11 | NA | 1 | NA | 5.03E-11 | 1.23E-11 | 3 | 9.1E-01 |
| **Y139C** | SCZ | 1.02E-08 | 1.67E-09 | 3 | **< 0.0001** | 3.00E-10 | 1.53E-10 | 3 | 2.9E-01 | 9.43E-11 | NA | 1 | NA | 7.71E-11 | 4.72E-11 | 2 | 1.0E+00 |
| **Q144E** | SCZ | 1.69E-09 | 6.97E-11 | 3 | 8.4E-01 | 1.43E-10 | 1.14E-10 | 3 | > 0.9999 | 3.84E-11 | NA | 1 | NA | 2.52E-11 | NA | 1 | NA |
| **R87Q** | Ctrl | 1.43E-09 | 1.42E-10 | 3 | 2.3E-01 | 2.38E-10 | 5.14E-11 | 3 | 4.9E-01 | 3.89E-11 | 1.54E-11 | 2 | 7.3E-01 | 2.71E-10 | NA | 1 | NA |
| **V109I** | Ctrl | 2.02E-09 | 1.44E-10 | 3 | 1.0E+00 | 1.91E-10 | 1.40E-10 | 3 | 9.6E-01 | 7.82E-11 | NA | 1 | NA | 2.78E-11 | 3.31E-12 | 2 | 3.1E-01 |
| **D123E** | Ctrl | 2.45E-09 | 9.13E-10 | 3 | 9.9E-01 | 2.45E-10 | 1.52E-10 | 3 | 6.0E-01 | 2.01E-10 | 1.41E-10 | 2 | 5.2E-01 | 2.59E-10 | 1.41E-10 | 2 | 1.4E-01 |
| **I131T** | Ctrl | 2.44E-09 | 6.40E-11 | 3 | 9.6E-01 | 3.31E-10 | 7.17E-11 | 3 | 1.5E-01 | 1.12E-10 | NA | 1 | NA | 1.25E-10 | 8.44E-11 | 3 | 8.9E-01 |
| **M146V** | Ctrl | 2.85E-09 | 3.25E-10 | 3 | 4.6E-01 | 2.65E-10 | 6.08E-11 | 2 | 5.2E-01 | 2.10E-10 | 1.40E-10 | 2 | 4.6E-01 | 1.34E-10 | 9.69E-11 | 2 | 8.9E-01 |

**Table S6: Calcium-dependent affinity of calmodulin towards Ca_V_1.2-IQ.** The K_D_ value for calmodulin’s affinity towards Ca_V_1.2-IQ at eight different calcium concentrations was determined from the data presented in Fig S9-S11. Statistical comparisons were performed at each calcium concentration using a one-way ANOVA followed by Dunnett’s *post hoc* test. Statistically significant differences are highlighted in bold and indicated with asterisks in Fig 5C-E. Statistical differences at high calcium levels (shaded in orange) are not shown, as the affinities here are very high and thus difficult to precisely interpret. *SD, standard deviation*.

| **Variant** | **Carrier phenotype** | **EC_50_ (M)** | **EC_50_ SD (M)** | **n** | ***P* value** | **Summary** |
| --- | --- | --- | --- | --- | --- | --- |
| **WT** |  | 4.79 E-07 | 2 .1 E-08 | 10 |  |  |
| **S82R** | SCZ | 6.53 E-07 | 5.9 E-08 | 3 | 0.015 | * |
| **R107C** | SCZ | 3.93 E-07 | 3.8 E-08 | 3 | 0.19 | ns |
| **I131L** | SCZ | 3.61 E-07 | 2.2 E-08 | 3 | 0.021 | * |
| **N138S** | SCZ | 3.53 E-07 | 2.0 E-08 | 3 | 0.013 | * |
| **Y139C** | SCZ | 7.79 E-07 | 4.3 E-08 | 3 | < 0.0001 | **** |
| **Q144E** | SCZ | 5.11 E-07 | 4.0 E-08 | 3 | 1.0 | ns |
| **R87Q** | Ctrl | 3.85 E-07 | 4.2 E-08 | 3 | 0.093 | ns |
| **V109I** | Ctrl | 6.23 E-07 | 5.4 E-08 | 3 | 0.075 | ns |
| **D123E** | Ctrl | 2.50 E-07 | 2.5 E-08 | 3 | 0.067 | ns |
| **I131T** | Ctrl | 4.23 E-07 | 2.7 E-08 | 3 | 0.63 | ns |
| **M146V** | Ctrl | 4.06 E-07 | 2.5 E-08 | 3 | 0.36 | ns |

**Table S7: EC_50_ values and statistics for Ca_V_1.2-IQ binding.** An EC_50_ model was fitted to the calcium-dependent CaM:Ca_V_1.2-IQ binding affinity curves presented in Fig 5B. Differences in the resulting EC_50_ values (Fig 5F) between WT and SCZ or control variants were determined with a one-way ANOVA followed by Dunnett’s *post hoc* test. *SD, standard deviation*.

| **Variant** | **Mean *f_300_*** | ***f_300_* SD** | **n** | ***P* value** | **Summary** |
| --- | --- | --- | --- | --- | --- |
| **WT** | 0.57 | 0.080 | 16 |  |  |
| **S82R** | 0.50 | 0.075 | 16 | 0.074 | ns |
| **R107C** | 0.56 | 0.086 | 6 | 1.0 | ns |
| **I131L** | 0.52 | 0.072 | 12 | 0.40 | ns |
| **Y139C** | 0.45 | 0.065 | 6 | 0.0093 | ** |
| **Q144E** | 0.59 | 0.099 | 6 | >0.99 | ns |

**Table S8: *f_300_* values and statistics for Ca_V_1.2 CDI.** The mean *f_300_* was calculated as the ratio of remaining calcium current to remaining barium current after 300 ms, based on the data presented in Fig 6B-G. Statistical differences between WT and variants were determined with a one-way ANOVA followed by Dunnett’s *post hoc* test. *SD, standard deviation*.

**Supplementary Figures**

**
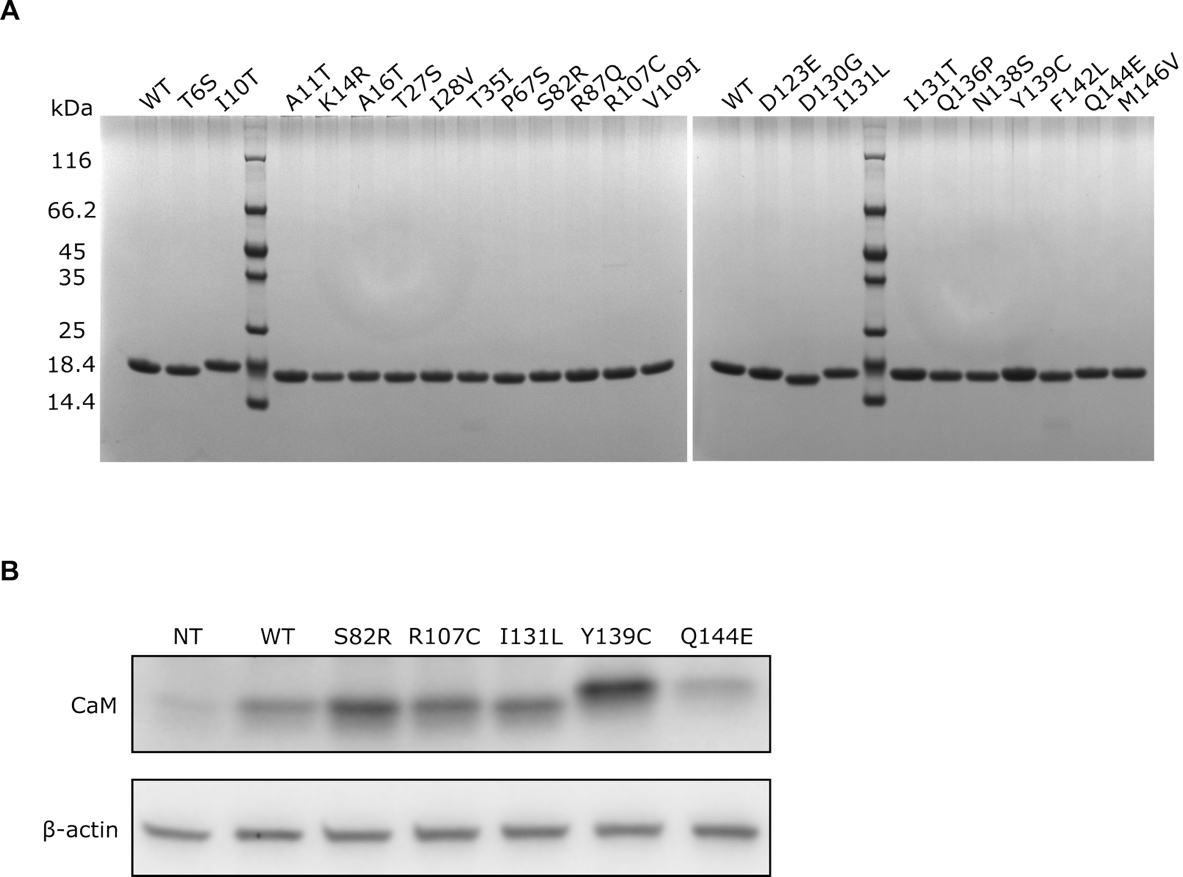
**

**Figure S1. A.** Coomassie-stained SDS-PAGE analysis of purified calmodulin protein. **B.** Western blot analysis of calmodulin expression levels in transiently transfected HEK293 cells. The blot is representative of three individual experiments. *NT, non-transfected*.

**
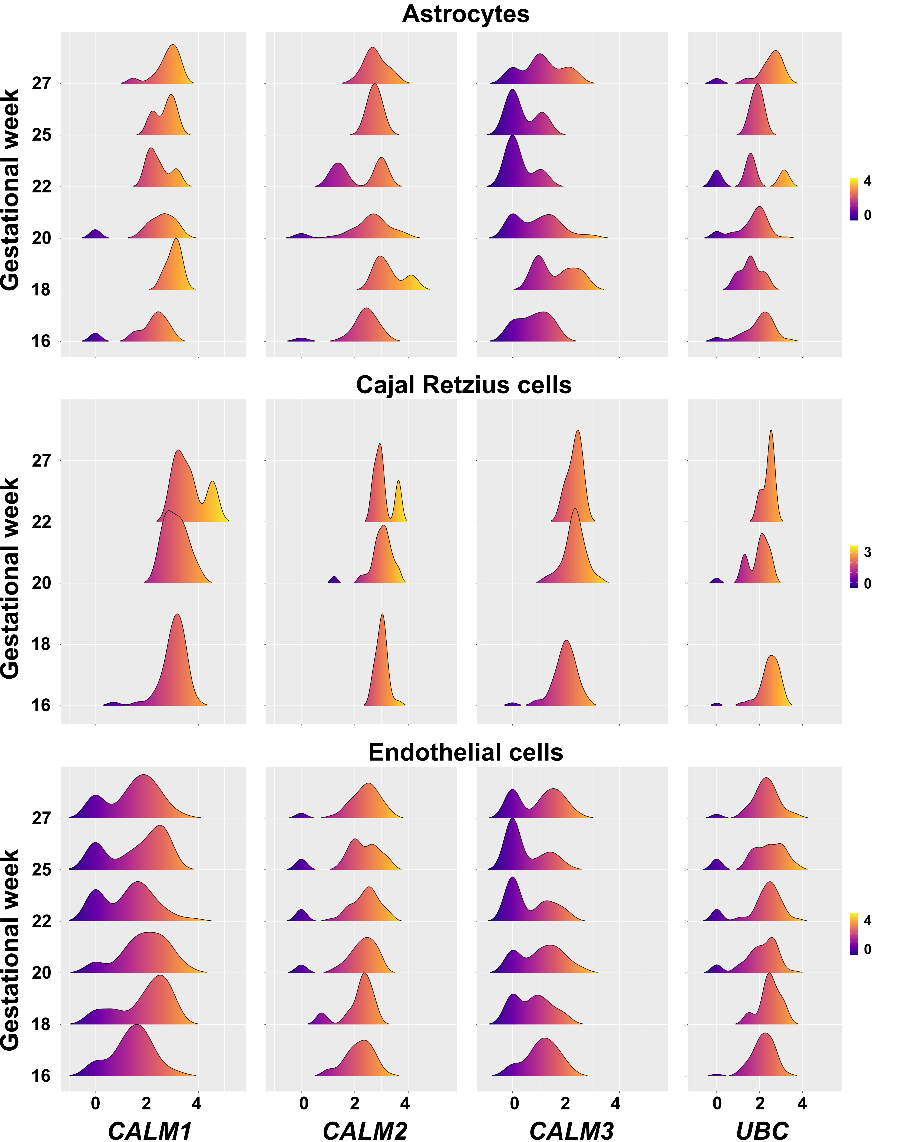
**

**Figure S2: Gene expression levels across gestational weeks.** Single-cell transcriptomics results for *CALM1*, *CALM2*, *CALM3*, and *UBC*, separated by gestational week for astrocytes, Cajal-Retzius cells, and endothelial cells. The expression levels of *CALM1*, *CALM2*, and *CALM3* are virtually identical across gestational weeks. The x-axis and color bars show scaled normalized expression levels, and the heights of the graphs represent cell counts.

**
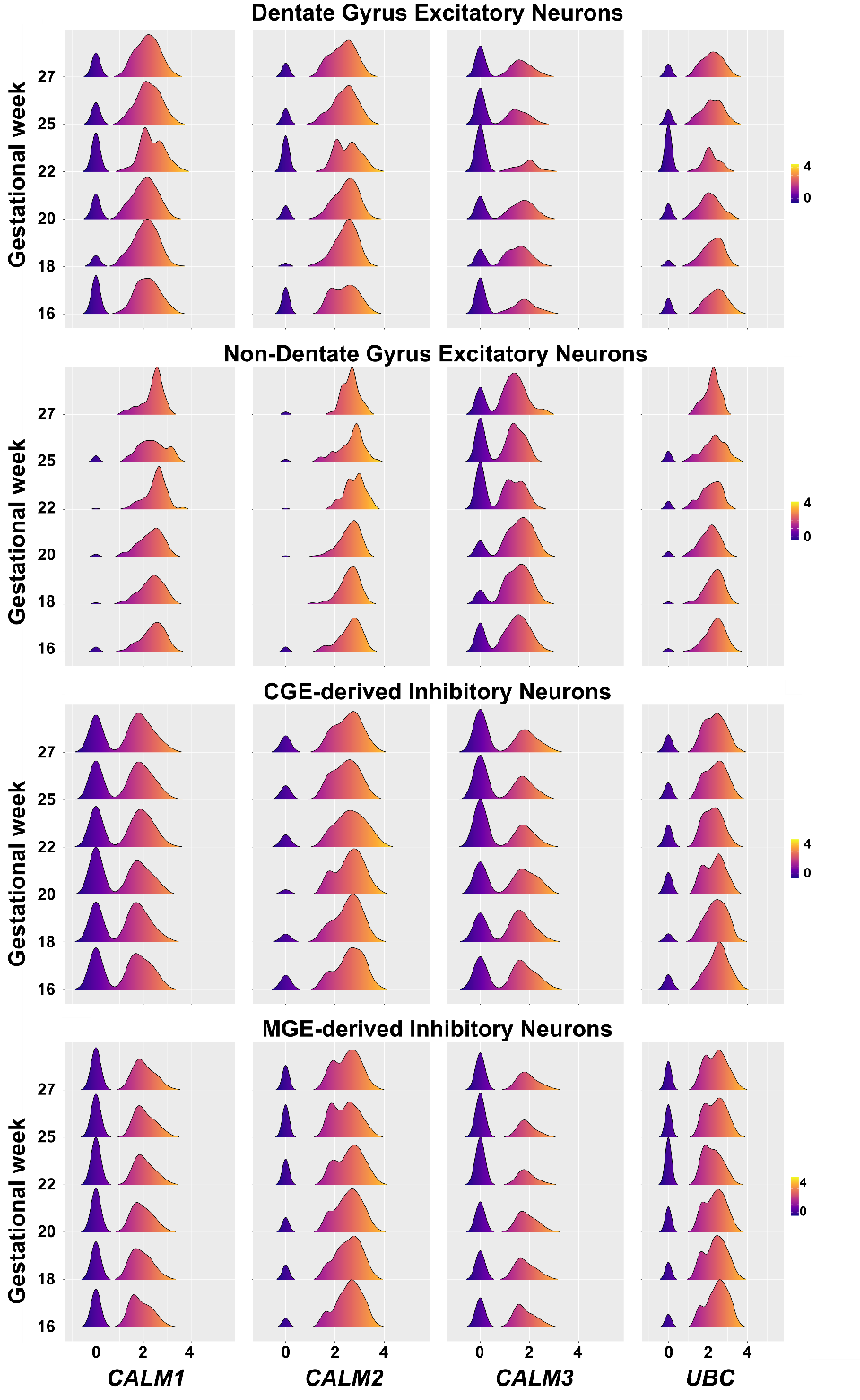
**

**Figure S3: Gene expression levels across gestational weeks.** Single-cell transcriptomics results for *CALM1*, *CALM2*, *CALM3*, and *UBC*, separated by gestational week for excitatory neurons in dentate gyrus, excitatory neurons not in dentate gyrus, caudal ganglion eminence (CGE)-derived inhibitory neurons, and medial ganglion eminence (MGE)-derived neurons. The expression levels of *CALM1*, *CALM2*, and *CALM3* are virtually identical across gestational weeks. The x-axis and color bars show scaled normalized expression levels, and the heights of the graphs represent cell counts.


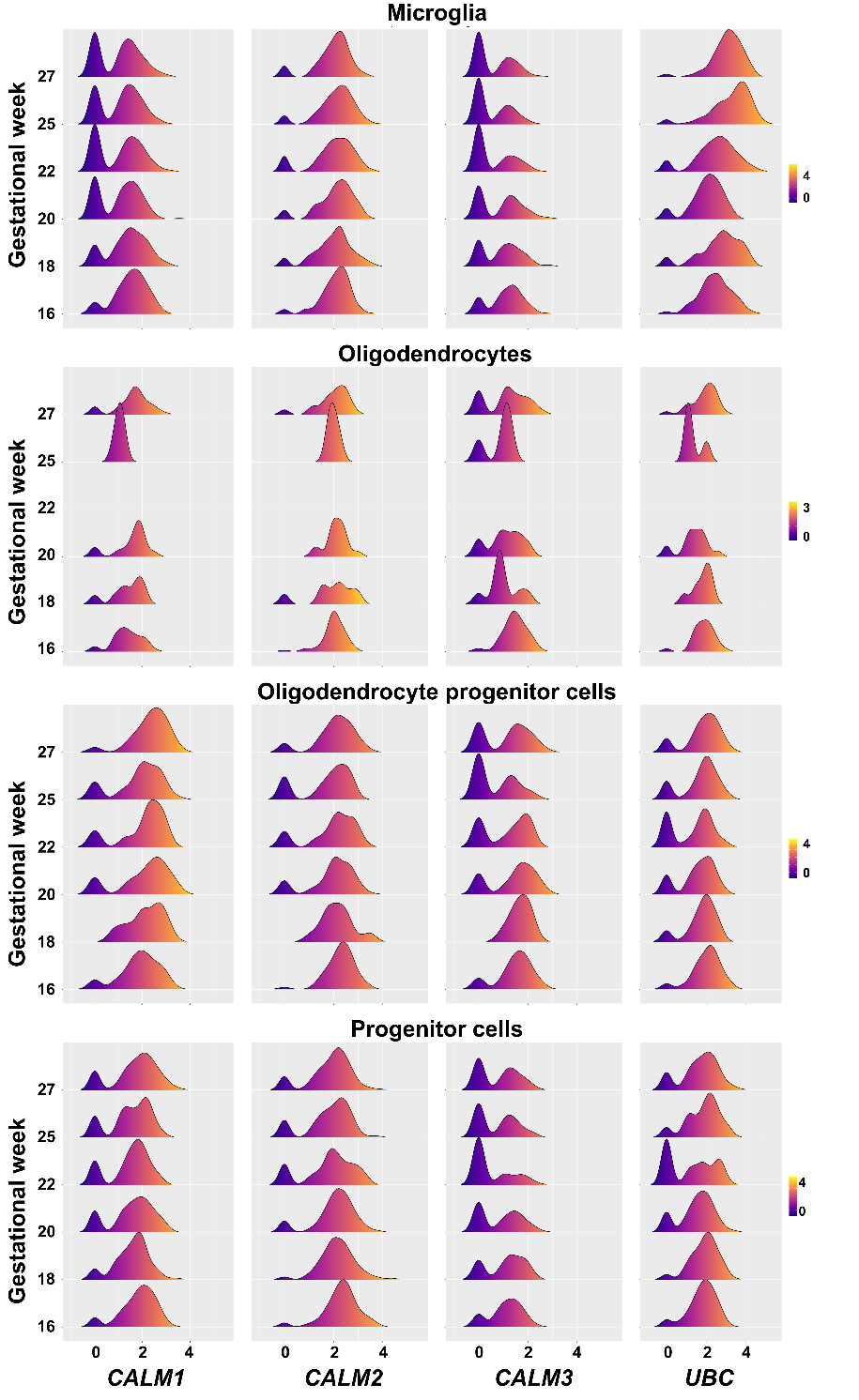


**Figure S4: Gene expression levels across gestational weeks.** Single-cell transcriptomics results for *CALM1*, *CALM2*, *CALM3*, and *UBC*, separated by gestational week for excitatory neurons in microglia, oligodendrocytes, oligodendrocyte progenitor cells (OPC), and in progenitor cells. The expression levels of *CALM1*, *CALM2*, and *CALM3* are virtually identical across gestational weeks. The x-axis and color bars show scaled normalized expression levels, and the heights of the graphs represent cell counts.


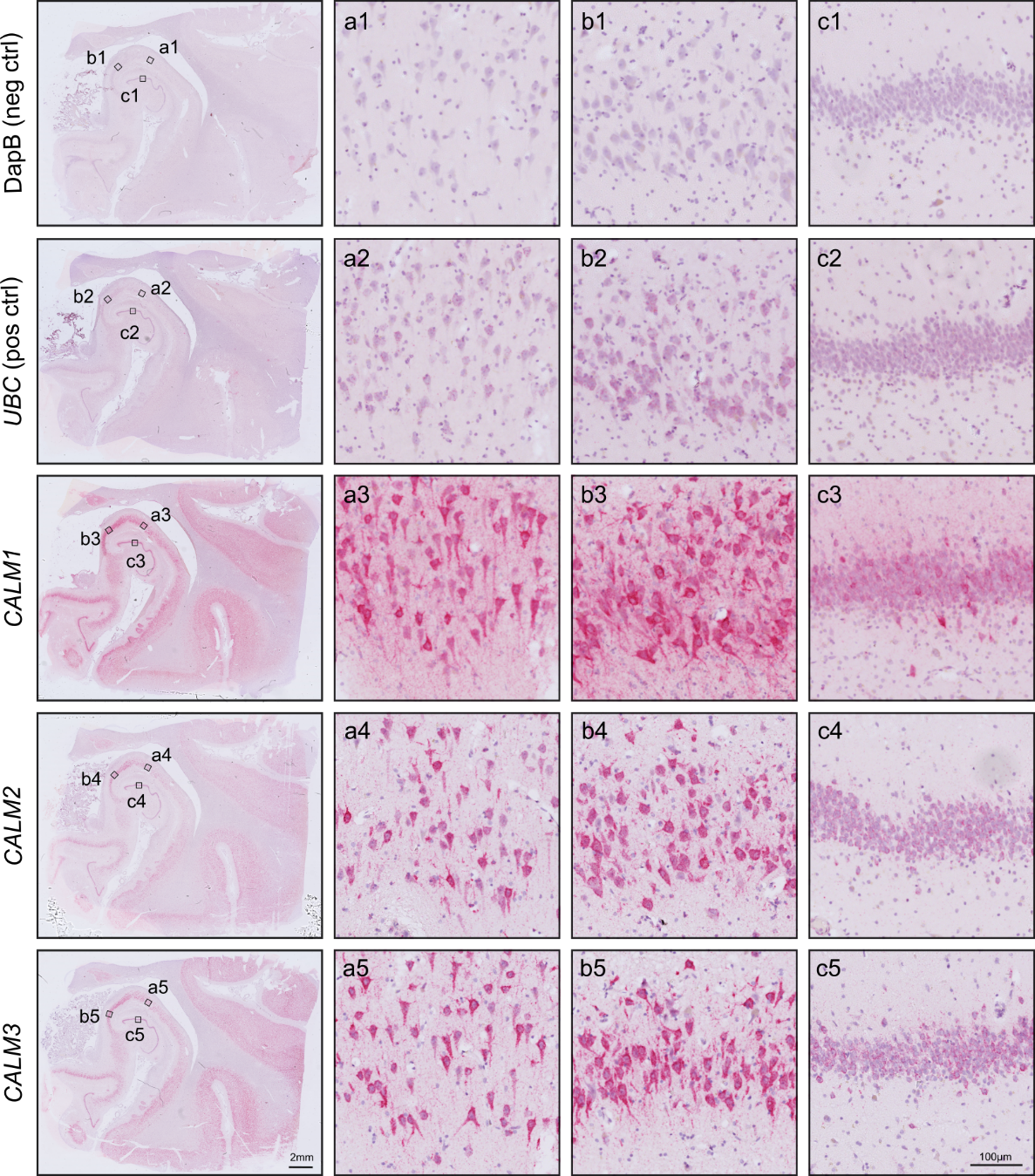


**Figure S5: *CALM1-3* expression in adult human hippocampus**. *In situ* hybridization with RNAscope probes (red signal) targeting the indicated genes of a healthy human donor (left). The depicted stains are representative examples of two donors – the other donor is shown in Fig 2C.


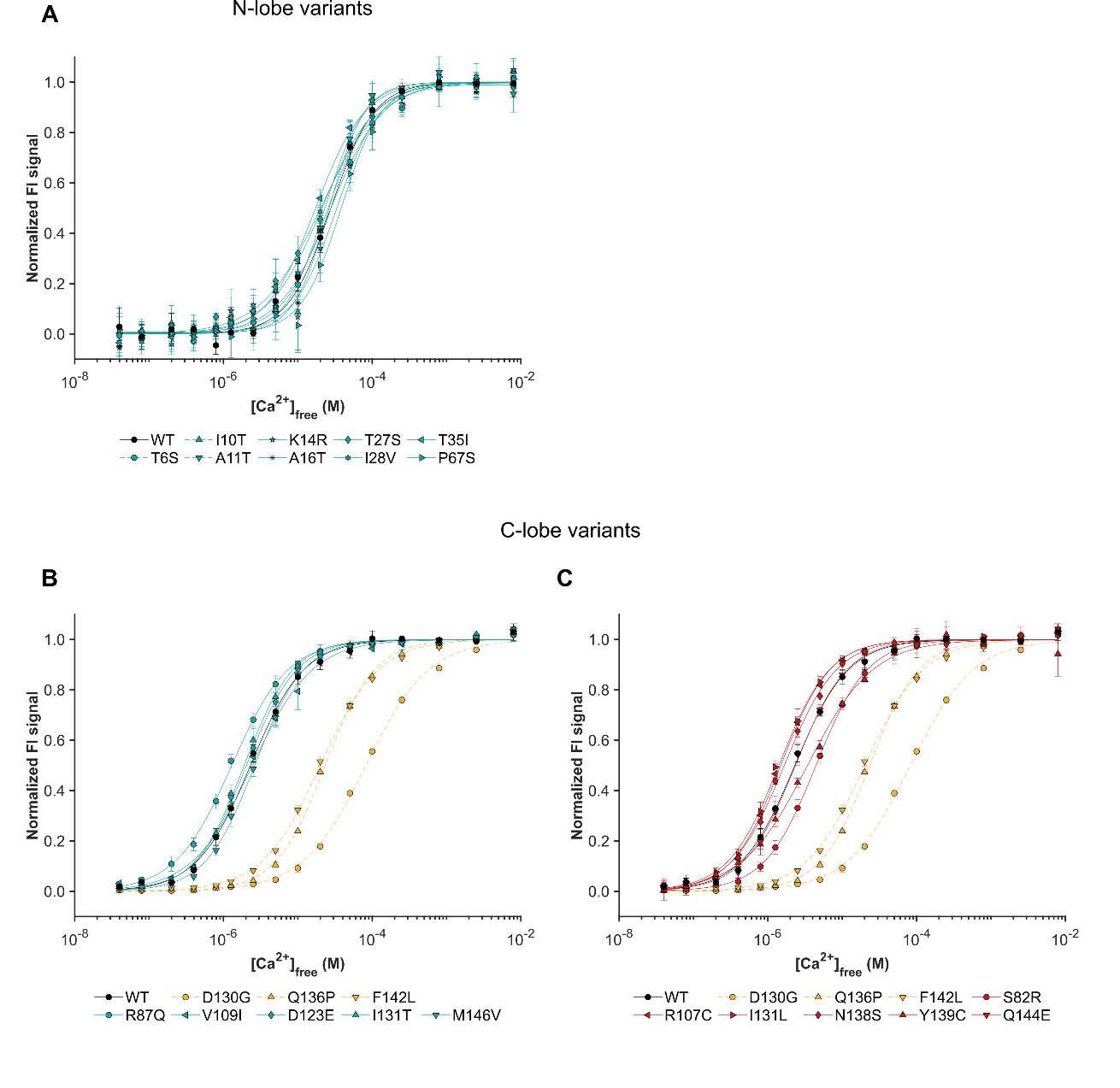


**Figure S6: Calcium binding curves for SCZ and control calmodulin variants.** **A.** Calcium binding curves of the indicated N-lobe calmodulin variants. **B-C.** Calcium binding curves of the indicated C-lobe calmodulin variants. Data normalization was only done for plotting purposes. All fitting was done on raw data. Yellow, LQTS; red, SCZ; teal, controls.


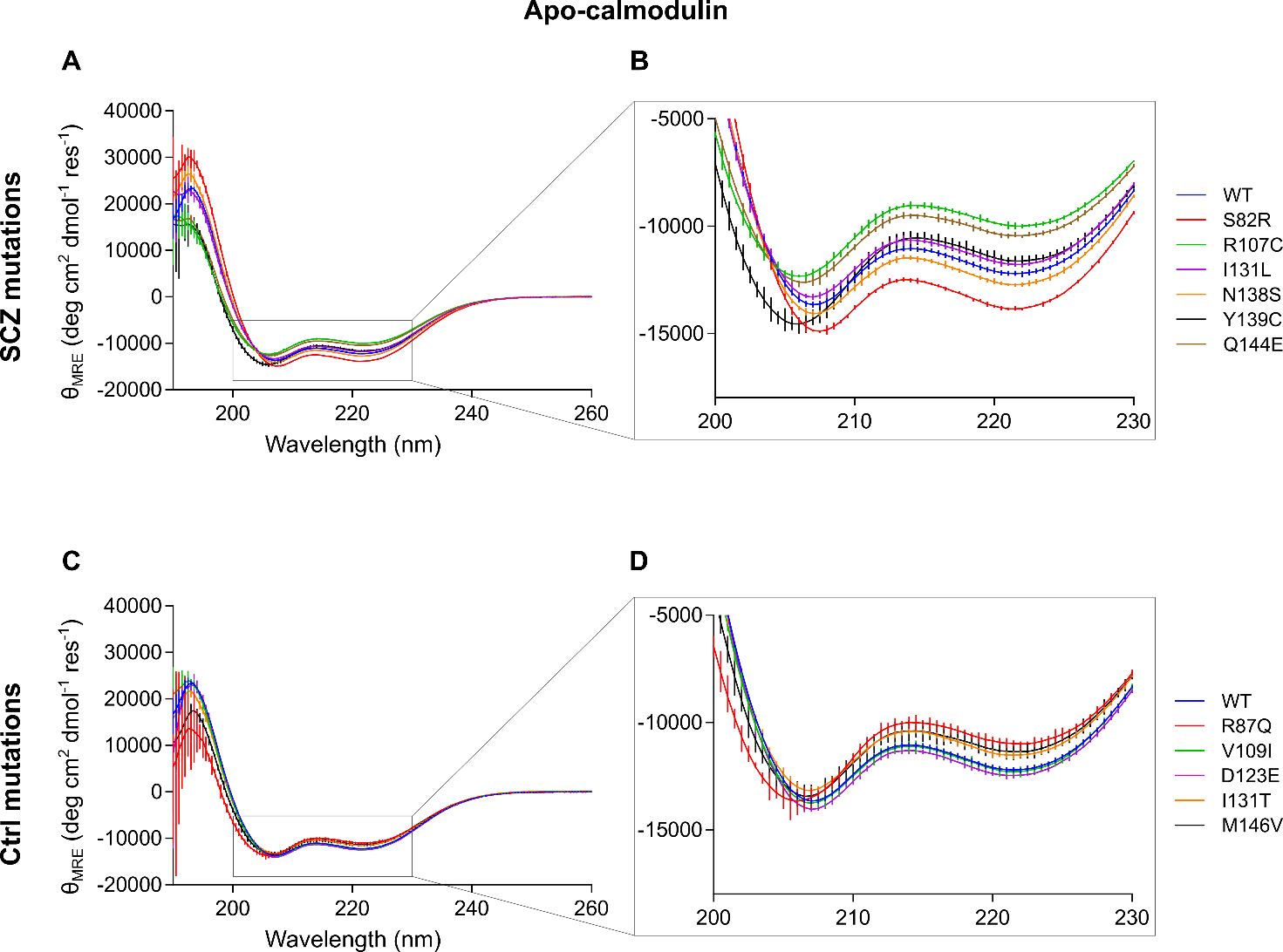


**Figure S7: Circular dichroism spectra of apo-calmodulin (calcium free). A-B.** Calmodulin with mutations from SCZ patients. **C-D.** Calmodulin with mutations from controls. For Fig 4 and Table S5, data points were extracted at 208 nm and 222 nm.


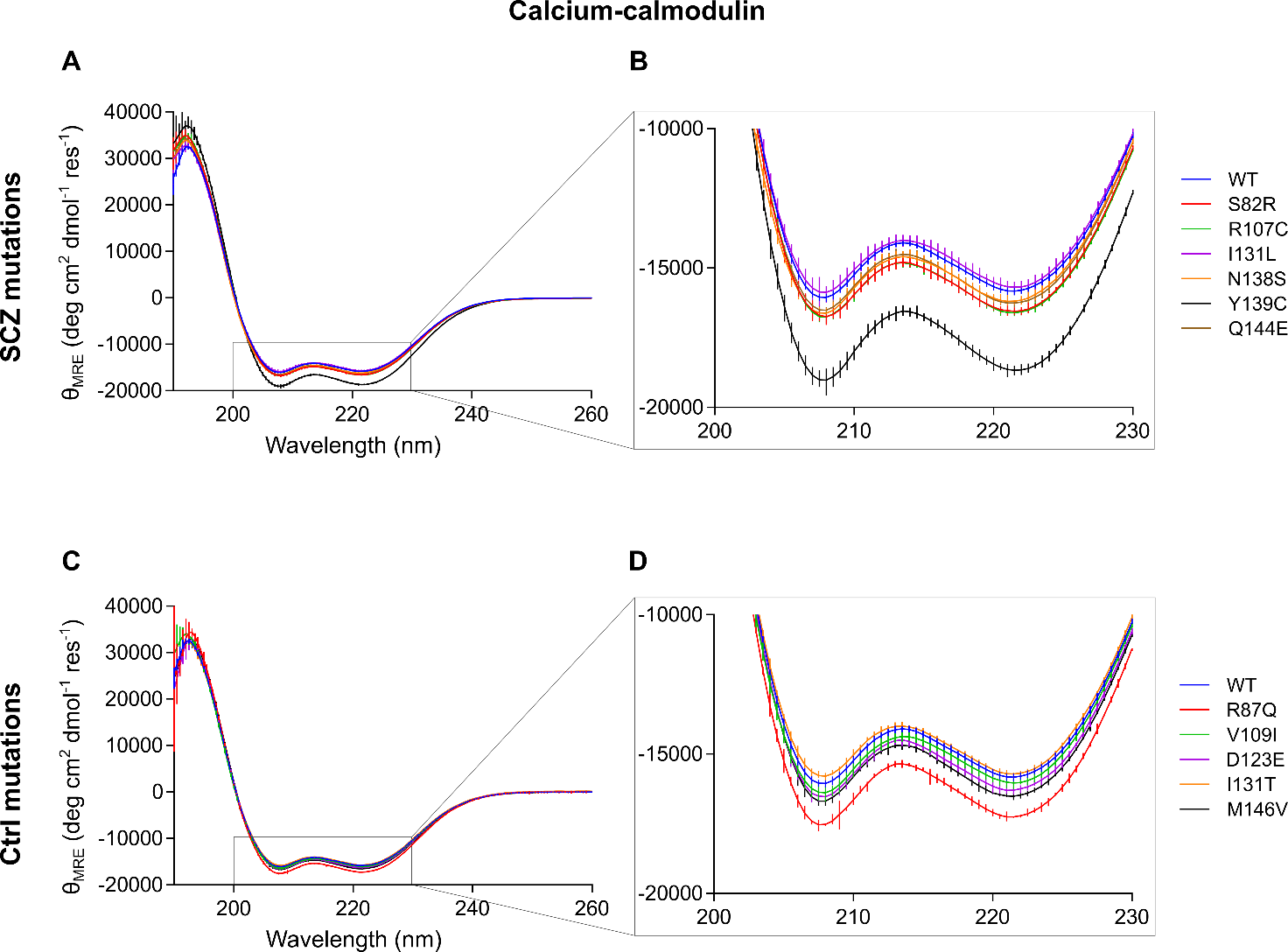


**Figure S8: Circular dichroism spectra of calcium bound calmodulin. A-B.** Calmodulin with mutations from SCZ patients. **C-D.** Calmodulin with mutations from controls. For Fig 4 and Table S5, data points were extracted at 208 nm and 222 nm.


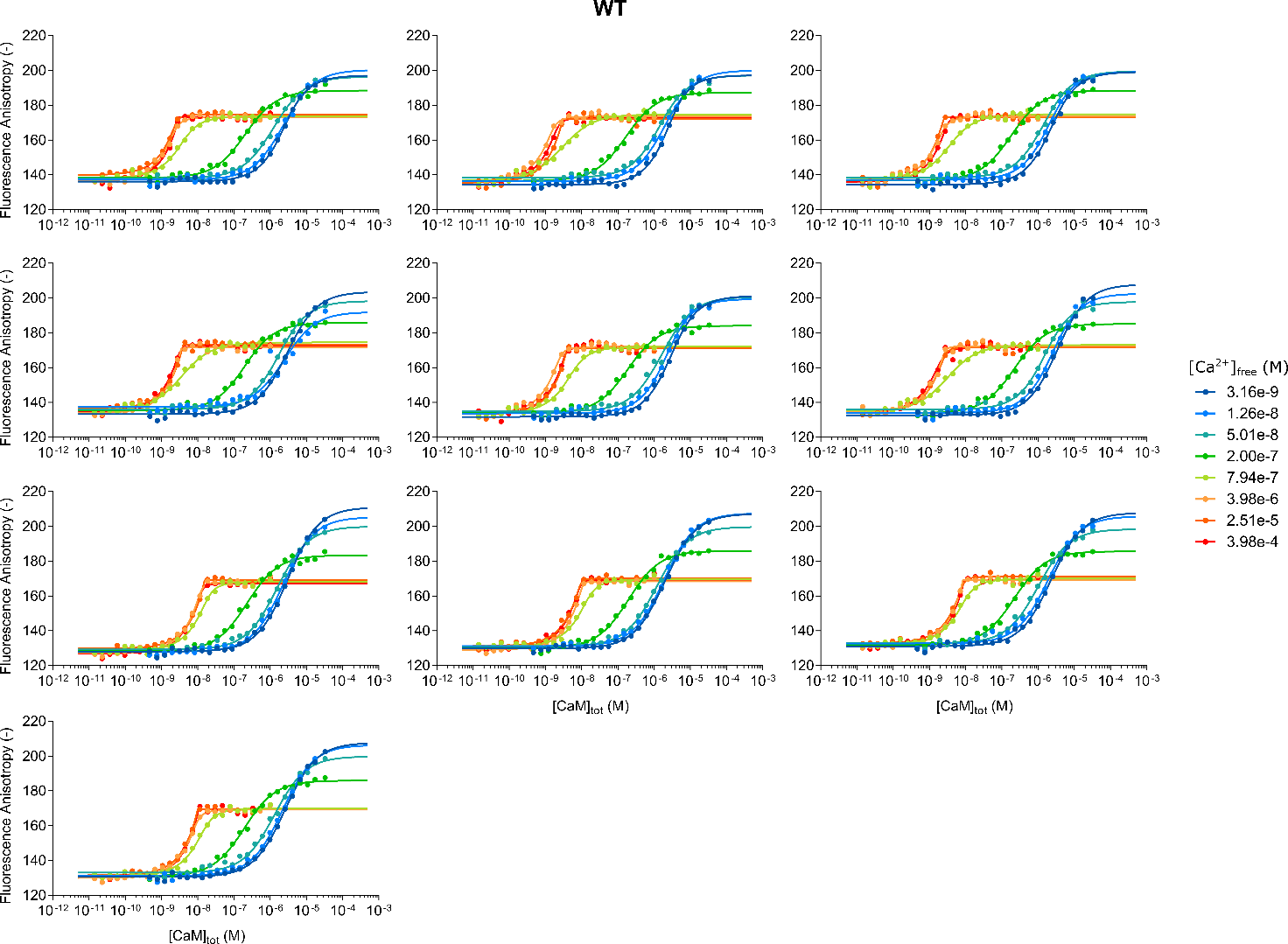


**Figure S9: Calcium-dependent binding of WT calmodulin to Ca_V_1.2.** Individual titration curves for WT calmodulin in the presence of Ca_V_1.2-IQ and eight different calcium concentrations. The plots show data from 10 independent experiments. A stoichiometric binding model was fitted to each binding curve, with resulting dissociation constants, K_D_ values, shown in Fig 5 and Table S6.


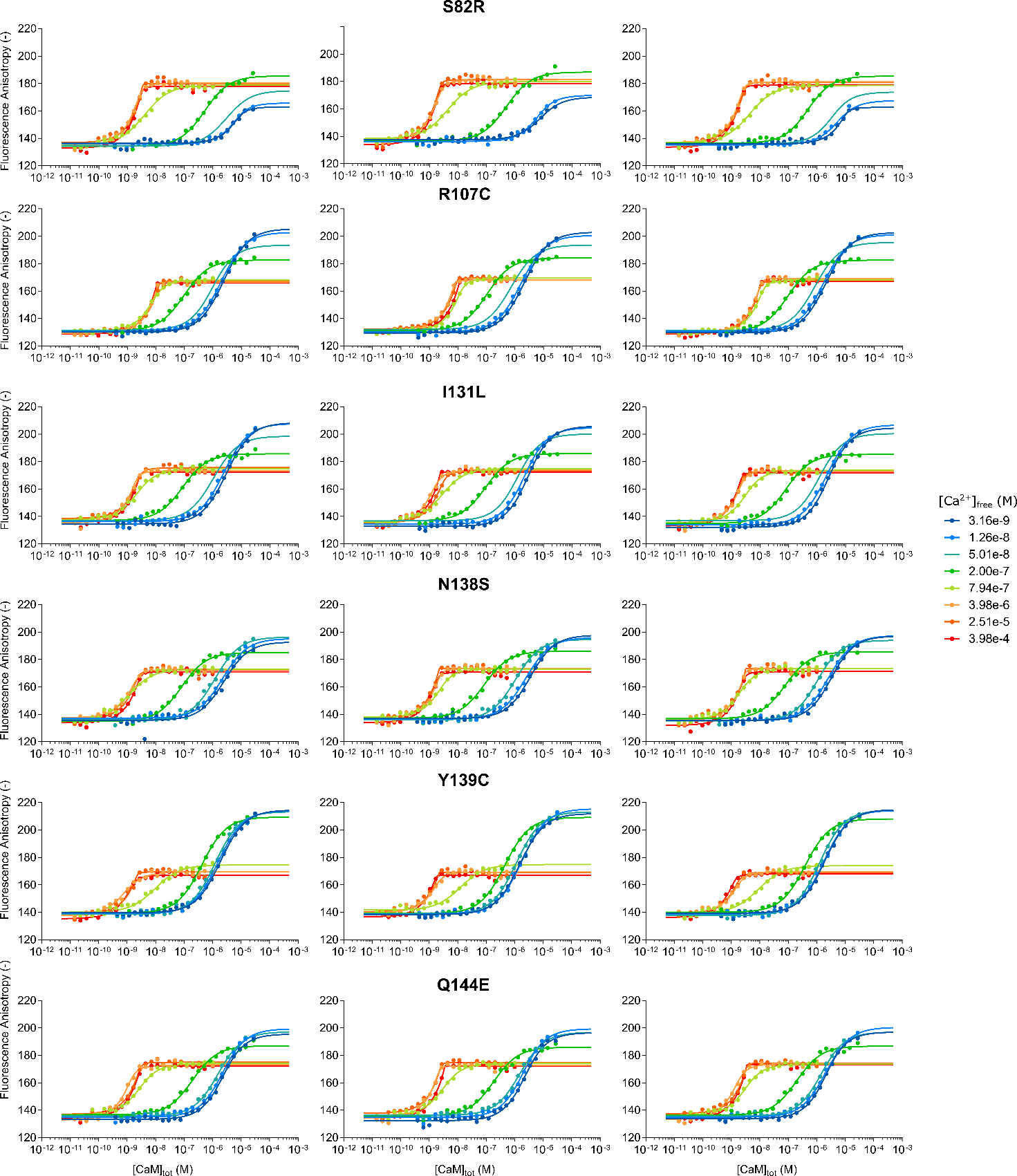


**Figure S10: Calcium-dependent binding of SCZ calmodulin variants to Ca_V_1.2.** Individual titration curves for calmodulin variants from SCZ patients in the presence of Ca_V_1.2-IQ and eight different calcium concentrations. The plots show data from three independent experiments for each variant. A stoichiometric binding model was fitted to each binding curve, with resulting dissociation constants, K_D_ values, shown in Fig 5 and Table S6.


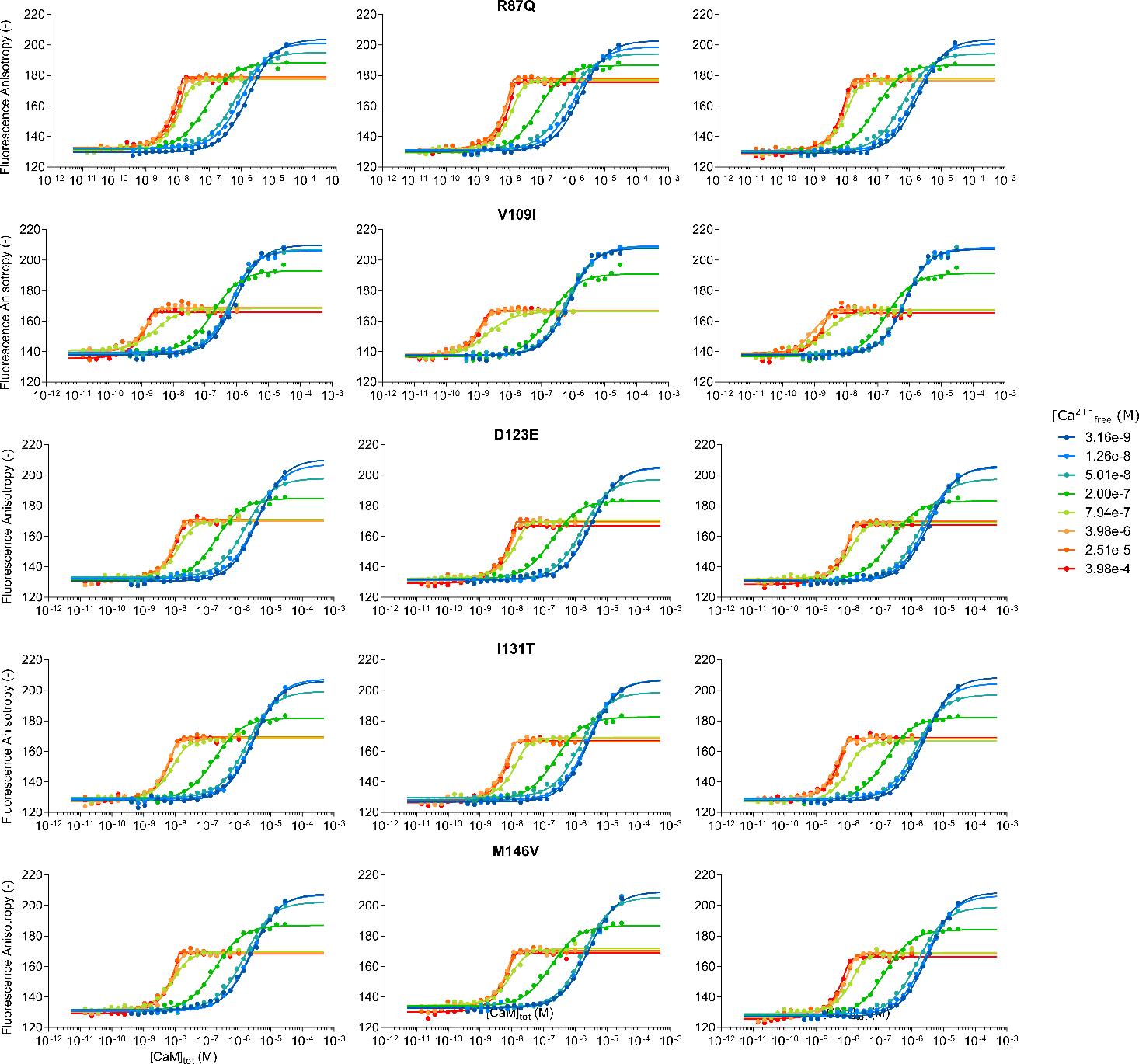


**Figure S11:** **Calcium-dependent binding of Ctrl calmodulin variants to Ca_V_1.2.** Individual titration curves for calmodulin variants from control individuals in the presence of Ca_V_1.2-IQ and eight different calcium concentrations. The plots show data from three independent experiments for each variant. A stoichiometric binding model was fitted to each binding curve, with resulting dissociation constants, K_D_ values, shown in Fig 5 and Table S6.


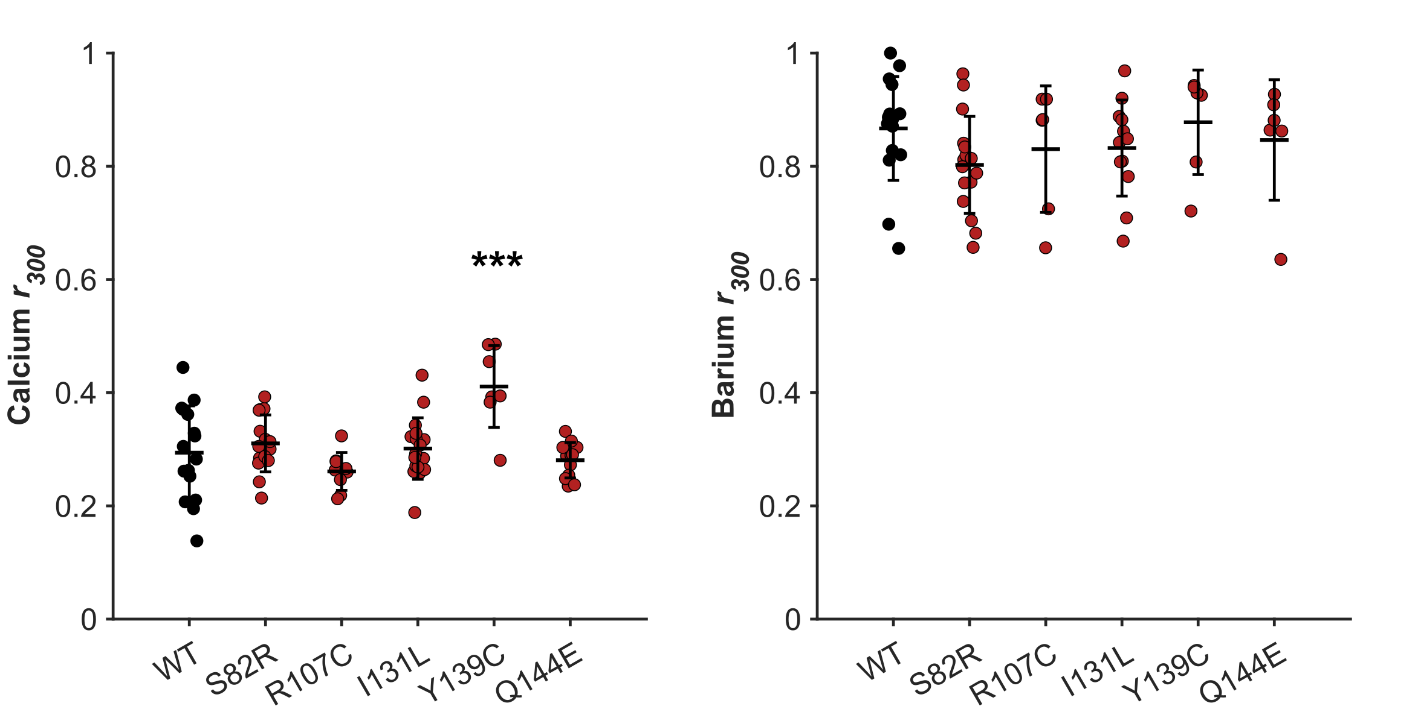


**Figure S12**: **Inactivation of Ca_V_1.2 by SCZ calmodulin variants.** The currents remaining 300ms (*r_300_*) after channel opening were determined for calcium as a measure of calcium-dependent inactivation (left) and for barium as a measure of voltage-dependent inactivation (right). n = 6-16 individual experiments, ***p<0.001 in one-way ANOVA with Dunnett’s *post hoc* test.
